# Unearthing phytochemicals as natural inhibitors for pantothenate synthetase in *Mycobacterium tuberculosis*: A computational approach

**DOI:** 10.3389/fphar.2024.1403900

**Published:** 2024-07-29

**Authors:** Mandeep Chouhan, Prashant Kumar Tiwari, Richa Mishra, Saurabh Gupta, Mukesh Kumar, Eman Abdullah Almuqri, Nasir A. Ibrahim, Nosiba Suliman Basher, Anis Ahmad Chaudhary, Vivek Dhar Dwivedi, Devvret Verma, Sanjay Kumar

**Affiliations:** ^1^ Biological and Bio-computational Lab, Department of Life Science, School of Basic Sciences and Research, Sharda University, Greater Noida, India; ^2^ Department of Computer Engineering, Parul University, Vadodara, Gujarat, India; ^3^ Department of Biotechnology, GLA University, Mathura, Uttar Pradesh, India; ^4^ Department of Biophysics, All India Institute of Medical Sciences, New Delhi, India; ^5^ Department of Biology, College of Science, Imam Mohammad Ibn Saud Islamic University (IMSIU), Riyadh, Saudi Arabia; ^6^ Center for Global Health Research, Saveetha Medical College and Hospitals, Saveetha Institute of Medical and Technical Sciences, Saveetha University, Chennai, India; ^7^ Bioinformatics Research Division, Quanta Calculus, Greater Noida, India; ^8^ Department of Biotechnology, Graphic Era (Deemed to be University), Dehradun, Uttarakhand, India

**Keywords:** tuberculosis, pantothenate synthetase, virtual screening, rutin, sesamin and catechin gallate

## Abstract

Pantothenate synthetase protein plays a pivotal role in the biosynthesis of coenzyme A (CoA), which is a crucial molecule involved in a number of cellular processes including the metabolism of fatty acid, energy production, and the synthesis of various biomolecules, which is necessary for the survival of *Mycobacterium tuberculosis* (*Mtb*). Therefore, inhibiting this protein could disrupt CoA synthesis, leading to the impairment of vital metabolic processes within the bacterium, ultimately inhibiting its growth and survival. This study employed molecular docking, structure-based virtual screening, and molecular dynamics (MD) simulation to identify promising phytochemical compounds targeting pantothenate synthetase for tuberculosis (TB) treatment. Among 239 compounds, the top three (rutin, sesamin, and catechin gallate) were selected, with binding energy values ranging from −11 to −10.3 kcal/mol, and the selected complexes showed RMSD (<3 Å) for 100 ns MD simulation time. Furthermore, molecular mechanics generalized Born surface area (MM/GBSA) binding free energy calculations affirmed the stability of these three selected phytochemicals with binding energy ranges from −82.24 ± 9.35 to −66.83 ± 4.5 kcal/mol. Hence, these identified natural plant-derived compounds as potential inhibitors of pantothenate synthetase could be used to inhibit TB infection in humans.

## Graphical Abstract

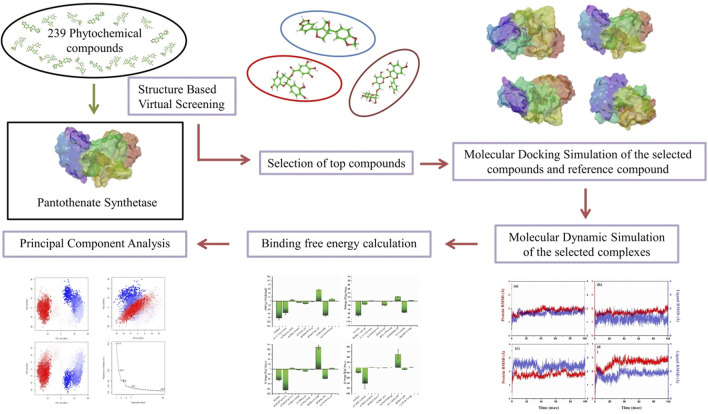

## Introduction

Tuberculosis (TB) is one of the deadliest infectious diseases caused by *bacillus Mycobacterium tuberculosis* (*Mtb*), which mainly affects human. Based on the Global Tuberculosis Report 2022, 10.6 million persons were diagnosed with TB in 2021, compared to 10.1 million infected persons in 2020. Additionally, the number of new cases of TB increased by 3.6% in 2021 as compared to 2020, which reversed the nearly 2% annual decline in TB cases that had been observed over the last two decades. Over 80% of TB cases and deaths occur in low- and middle-income nations. As per the WHO, in 2022, the new cases mostly emerged in South-East Asian region (46%), followed by Africa (23%) and the Western Pacific (18%). Approximately 87% of new cases are found mainly in high-burden countries, including Bangladesh, China, Democratic Republic of the Congo, India, Indonesia, Nigeria, Pakistan, and the Philippines. Globally, nearly half of TB patients and their families suffer with financial burden that costs approximately 20% of the total household income, which undermines the WHO policy for the complete eradication of TB. Individuals with compromised immune systems, such as human immunodeficiency virus (HIV), undernutrition, or diabetes, or those who smoke, may face elevated risks of TB. In 2022, undernutrition accounted for 2.2 million new TB cases globally, followed by HIV infection (0.89 million), alcohol use disorders (0.73 million), smoking (0.70 million), and diabetes (0.37 million) (Bagcchi, 2023).

TB has been characterized as a substantial public health concern worldwide, as existing medications are limited due to the serious side effects, prolonged therapy, and the rise of multi-drug resistance TB (MDR-TB) and extensively drug resistance TB (XDR-TB) (Malothu et al., 2018; Bhagwat et al., 2022). The development of new drugs for the treatment of TB has gained scientific attention in recent years. The main issue in treating this disease is the increase in the number of persons infected with resistant *Mtb* strains such as MDR-TB (Seung et al., 2015; Gygli et al., 2017; Marchetti et al., 2018). Drug-resistant TB is a dangerous type of TB that has evolved over the past few decades, when bacteria become resistant to the drugs used to treat the disease (Uddin et al., 2019). Recently, there have been two drug regimens available for TB treatment: first-line drugs (rifampicin, isoniazid, pyrazinamide, and ethambutol), which are the core treatment regimens, and second-line drugs, such as fluoroquinolones (levofloxacin or moxifloxacin) and injectables (amikacin, capreomycin, and kanamycin), which are prescribed for drug-resistant TB (Singh et al., 2020). However, these drugs produce severe adverse effects to humans. Hence, there is a need to develop potential new medications against *Mtb* targets, which may produce minimal side effects to humans. As per the TB Structural Genomics Consortium (TBSGC), more than 400 different potential *Mtb* targets have been identified from the *Mtb* genome. Out of the 185 different targets from diverse biochemical pathways, 16 have protein database structures, and 102 are in various stages of development at the TBSGC. Furthermore, the understanding of the key stages of development and alternate biosynthetic pathways during non-replicating persistent *Mtb* has led to the identification of over 200 prospective targets (Suresh et al., 2020). Among them, pantothenate synthetase was one of the potential drug targets of *Mtb* (Hassan et al., 2020).

The pantothenate biosynthesis pathway has been suggested as a potential pharmacological target to develop novel medications for the treatment of TB (Suresh et al., 2020). This pathway is more intriguing because it is not present in mammalian cells, but it is necessary for *Mtb*’s virulence and persistent proliferation (Smith, 2003; Narender et al., 2016; Lunghi et al., 2022). There are four steps involved in the biosynthesis of pantothenate, which is processed by enzymes produced by the genes panB, panC, panD, and panE (Suresh et al., 2020). Pantothenate synthetase is encoded by panC (Devi et al., 2015), which catalyzes the ATP-dependent condensation of D-pantoate and β-alanine to generate pantothenate through the formation of an intermediate, pantoyl adenylate, which is the final step of pantothenate biosynthesis (Arokia Rajan et al., 2023). Pantothenate (vitamin B5) is a significant precursor of coenzyme A (CoA) and acyl carrier protein (Hassam et al., 2023), which are required for a variety of intracellular processes such as the synthesis of polyketides and non-ribosomal peptides, as well as the metabolism of fatty acids and cell signaling (Narender et al., 2016; Goud et al., 2017). Mammals obtain pantothenate from their diet, as there is no biosynthetic pathway for it, whereas microorganisms synthesize it (Goud et al., 2017).

Some of the well-known pantothenate synthetase protein inhibitors including nafronyl oxalate (White et al., 2007), 5-tertbutyl-N-pyrazol-4-yl-4,5,6,7-tetrahydrobenzo[d]isoxazole-3- carboxamide derivatives (Velaparthi et al., 2008), the chemical class of 3-biphenyl-4-cyanopyrrole-2-carboxylic acids (Kumar et al., 2013), the analogs of 3-phenyl-4,5,6,7-tetrahydro-1H-pyrazolo[4,3-c]pyridine (Samala et al., 2013), 2-(2-(benzofuran-2-yl-sulfonylcarbamoyl)-5-methoxy-1H-indol-1-yl) acetic acid (Samala et al., 2014), (E)-2-hydroxy-5-((4-(N-(2-oxobut-3-en-1-yl)sulfamoyl)phenyl)diazenyl)benzoic acid (Pradhan and Sinha, 2018), nimocinolide (Mahanta et al., 2021), fucoidan and kappa carrageen (Arokia Rajan et al., 2023), and pyrazolo[3,4-*b*]pyridine with N(1)CH_3_, C(3)C_6_H_5_, C(4) *p*CH_3_C_6_H_5_, C(5)CO_2_Et, C(6)SMe substitutions (Rao et al., 2024) have been identified. These studies consistently revealed the importance of pantothenate synthetase in *Mtb*. Despite these, there is a need to find potential plant-derived natural compounds, which could inhibit *Mtb* pantothenate synthetase with better binding efficiency.

Plants have played a diverse role in supplying necessities like food, medicine, clothing, and shelter. There has been thorough exploration of natural compounds for uncovering new pharmaceuticals (Nagoor Meeran et al., 2021). For many decades, plants have been reservoirs of antibiotics, anticancer substances, analgesics, and cardioprotective agents, among other medicinal application (Subhedar et al., 2017; Singh et al., 2023; Chunarkar-Patil et al., 2024). In the current work, we used a phytochemical library of 239 compounds from various medicinal plants to perform virtual screening with the aim of identifying possible phytochemical inhibitors of *Mtb* by targeting the active site of protein pantothenate synthetase.

## Materials and methods

### Compilation of compounds and receptor

A 3D crystal structure of pantothenate synthetase in association with AMPCPP, pantoate, and a compound that serves as a reaction intermediate, pantoyl adenylate (PDB ID: 1N2E), solved at resolution 1.6 Å was downloaded from the PDB database for structure-based virtual screening (SBVS) (https://www.rcsb.org/) (Berman et al., 2000). A library was prepared using 97 phytochemical compounds from this paper (Barbieri et al., 2017), demonstrating that these phytochemicals may be a possible source of effective, cheap, and safe antimicrobial agents with lower adverse and side effects. So, these phytochemicals were downloaded from the PubChem database (Supplementary Table S1). Additional 142 antibacterial phytochemicals from spices, herbs, and some other plants were also included (Supplementary Table S2). Therefore, the library of total 239 compounds was generated using Progenesis SDF studio (Nonlinear, 2023).

### Protein and ligand preparation, active site residue prediction, and structure-based virtual screening

To prepare the target protein for SBVS, a series of essential steps were taken to ensure its suitability for virtual screening. These steps are commonly followed in molecular docking studies to optimize the target protein for ligand binding analysis. The steps involved the removal of native ligands, solvent, and ions, followed by assigning Gasteiger charges, removal of non-polar hydrogen atoms, and addition of polar hydrogen atoms using default settings of the Dock Prep tool in UCSF Chimera (Pettersen et al., 2004; Trott and Olson, 2010). After that, using computed atlas of surface topography of proteins (CASTp), some specific residues (Pro^38^, Thr^39^, Met^40^, Gly^41^, His^44^, Gly^46^, His^47^, Ala^49^, Leu^50^, Ser^65^, Phe^67^, Asn^69^, Met^71^, Gln^72^, Phe^73^, Gly^74^, Asp^78^, Ala^81^, Tyr^82^, Pro^83^, Leu^127^, Glu^128^, Arg^132^, Thr^134^, His^135^, Phe^136^, Val^139^, Val^142^, Val^143^, Leu^146^, Phe^156^, Phe^157^, Gly^158^, Lys^160^, Asp^161^, Tyr^162^, Gln^163^, Gln^164^, Val^184^, Pro^185^, Thr^186^, Val^187^, Met^195^, Ser^196^, Ser^197^, Arg^198^, Tyr^201^, Tyr^249^, Leu^269^, Val^270^, Thr^276^, Thr^277^, Arg^278^, Leu^280^, Asp^281^, and Asn^282^) were selected as active site residues in pantothenate synthetase protein for SBVS (Tian et al., 2018). Furthermore, the Gasteiger partial charge was added to the library of phytochemical compounds followed by the energy minimization via the universal force field using default settings in the PyRx, which is a virtual screening tool (Rappe et al., 1992). The prepared library of 239 phytochemical compounds was virtually screened against pantothenate synthetase using Pyrx (Dallakyan and Olson, 2015). The compounds were evaluated on the basis of their binding energy, with the top 10 phytochemical compounds selected due to their ability to form the most favorable interactions with the protein.

### Redocking, molecular interaction analysis, and ADME profiling

AutoDock Vina plugin in Chimera was employed for molecular docking simulations between the pantothenate synthetase and top 10 selected phytochemicals in order to identify the residues that interact with reference molecules the most. So, both the target protein and ligand for all the complexes have been prepared for docking using default parameters of Dock Prep tool in Chimera. At the ligand binding site in the pantothenate synthetase protein, molecular docking was lastly carried out using AutoDock Vina plugin using default settings. This was done by changing the grid size to 35.54 × 32.31 × 44.51Å at axes (X, Y, and Z), which covers all the necessary residues in the area of 39.02 × 27.79×33.12 region, in order to provide sufficient area for ligand conformations during the docking process. Here, top three docked complexes with the highest negative docking score were chosen, which were shown to have the same binding affinity as SBVS. These protein–ligand complexes were then minimized using the structure minimization tool in UCSF Chimera to put the 3D protein structure of pantothenate synthetase and possible phytochemical as inhibitors to minimization (Pettersen et al., 2004). Following that, SwissADME was used to perform additional absorption, distribution, metabolism, and excretion (ADME) studies on the top three phytochemicals showing the greatest negative docking scores (Daina et al., 2017; Chouhan et al., 2023). At last, Maestro v12.8 tool in Schrodinger suite was used to create all of the 3D and 2D interaction visuals (Schrödinger, 2024a). A similar docking protocol was used for the diphosphomethylphosphonic acid adenosylester ligand against pantothenate synthetase, which acts as a reference complex to compare the top three complexes.

### Molecular dynamics simulations

The molecular dynamics (MD) simulation of top three protein–ligand docked complexes obtained from docking was performed to analyze the dynamic stability and intermolecular interactions at 100 ns using the academic package of Desmond–Maestro 2020–4 (D. E. Shaw Research, 2021; Schrödinger, 2021; Bowers et al., 2006). MD systems for all docked complexes were created as orthorhombic grid boxes (20 Å × 20 Å × 20 Å buffer), and then transferable intermolecular potential four-point water molecules were added to minimize this system, which was further neutralized by the addition of counter Na^+^ and Cl^−^ ions when placed at 20 Å distance around the docked compound present in the active site of pantothenate synthetase. Additionally, to reserve the constant pressure during simulation, a 0.002 ps time interval was selected for the anisotropic diagonal position scaling. In addition, the temperature of the system was adjusted to 300 K coupled with a 20 ps normal temperature and pressure (NPT) reassembly at 1 atm pressure. Additionally, the density of the system was kept close to 1 g/cm3, and the whole calculations were performed using default settings. At last, MD simulation for all the selected docked complexes was run for 100 ns intervals under the same circumstances. The complete data calculation was carried out using the OPLS 2005 force field for all atoms (Harder et al., 2016). RMSD, RMSF, and protein–ligand interaction profiling were measured using MD simulation trajectory for every putative docked compound, which form a complex with pantothenate synthetase.

### Molecular mechanics generalized Born surface area calculations

The calculation of binding free energy of protein–ligand complexes was carried out by performing molecular mechanics generalized Born surface area calculations (MM/GBSA) on the final 10 poses, taken at regular intervals of 10 ns from the corresponding MD simulation trajectory using the default settings in the Prime MM/GBSA module in Schrodinger’s suite (Jacobson et al., 2004; Schrödinger, 2024b). This method was carried out using docked complexes and poses derived from MD simulation trajectories, whereas explicit ions and transferable intermolecular potential four-point (TIP4P) water molecules were retrieved from the selected complex for refinement before performing the MM/GBSA calculation (Ntie-Kang et al., 2014).

### Principal component analysis

Using the Desmond–Maestro interpolarity tool, the simulation interaction diagram was analyzed to investigate simulation trajectories. For each complex studied, a MD trajectory was obtained and converted into a Bio3D-compatible format. Employing "R" programming language, principal component analysis (PCA) was then performed to analyze the dynamics of the systems (Grant et al., 2006; Li, 2021). Furthermore, the resultant trajectories, featuring coordinates recorded at intervals of every 20 ps, were subjected to evaluation using the simulation interaction diagram feature within the Schrödinger package. These simulations yielded essential metrics such as RMSD, RMSF, and the profile of protein–ligand interactions. These analyses provided valuable insights into the structural stability, flexibility, and dynamics of each complex over a 100 ns MD simulation. Additionally, post-dynamics calculations were carried out using the Bio3D tool, enabling research workers to perform further analyses and gain deeper insights into the behavior of the studied systems. The combination of the Desmond–Maestro interpolarity tool, the "R" program, and the Schrödinger package, along with the Bio3D tool, offered a comprehensive approach to understand the dynamics and interactions within the protein–ligand complexes under investigation.

## Result and discussion

### Structure-based virtual screening

**FIGURE 1:**
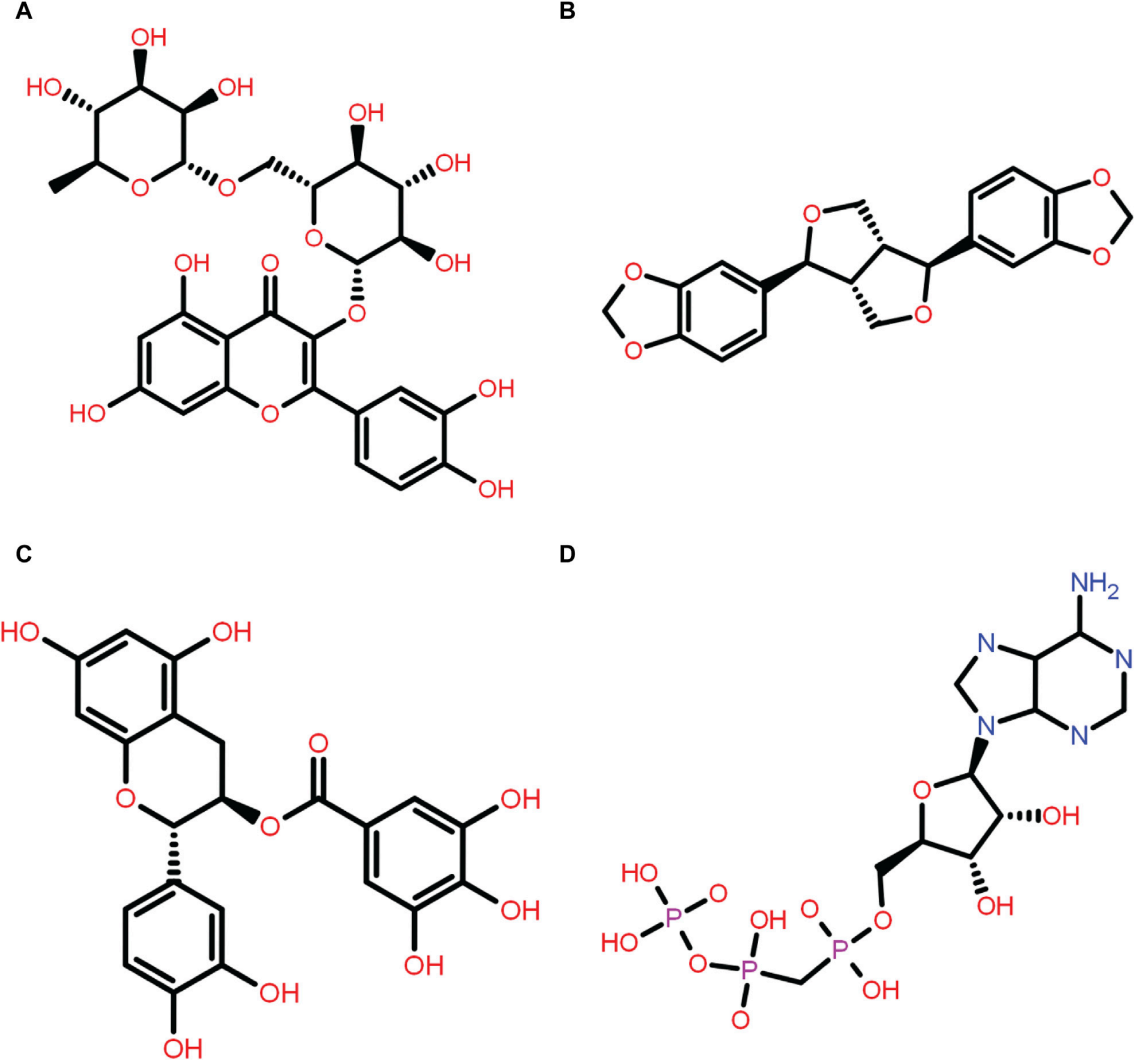
Two-dimensional structure of the selected phytochemical compounds and the reference molecule, that is, **(A)** rutin, **(B)** sesamin, **(C)** catechin gallate, and **(D)** diphosphomethylphosphonic acid adenosylester.

The main objective of this research was to identify potential candidate from a natural source that can inhibit pantothenate synthetase to treat TB. Thus, a prepared library of 239 phytochemical compounds with antibacterial properties (Supplementary Tables S1, 2) belonging to the spices, herbs, and some other plants was used for SBVS against the pantothenate synthetase protein. This resulted in a variety of compounds with docking scores ranging from −1.8 kcal/mol to −11 kcal/mol against the pantothenate synthetase protein (Supplementary Table S3). Subsequently, prioritizing compounds according to their binding energy, exclusively the foremost 10 phytochemicals, that is, rutin, theaflavine, sesamin, proanthocyanidins, catechin gallate, smilagenin, gitogenin, diosgenin, 4′-O-methylglabridin, and 1,2,6-tri-O-galloyl-beta-d-glucopyranose, were selected as bispecific inhibitors for subsequent redocking and intermolecular interaction study in contrast with control compound diphosphomethylphosphonic acid adenosylester inhibitor against pantothenate synthetase (Figure 1D). Herein, the selected 10 phytochemical compounds showed significant docking scores ranging from −10.2 to *−*11 kcal/mol against the protein pantothenate synthetase (Supplementary Table S4).

### Redocking simulation and intermolecular interaction analysis

In order to further validate the binding energy score derived from the SBVS result, the top 10 selected phytochemical compounds were redocked (Supplementary Table S4). Of these, the top three compounds that displayed the same docking score were chosen: rutin, sesamin, and catechin gallate (docking score range in between −11 and −10.3 kcal/mol) (highlighted with the bold text in Supplementary Table S4), and then intermolecular interaction analysis was conducted on them (Table 1). The scoring function of SBVS was used to predict the compounds, which shows optimal conformation and stability against the protein pantothenate synthetase. These predictions underwent validation through redocking and intermolecular interaction analysis. Redocking was performed for all the three selected phytochemicals in the binding pocket of pantothenate synthetase to find the binding poses with the best fit and stability. A lower RMSD value indicates a better fit between the two sets of coordinates, whereas a lower docking score indicates stronger binding affinity. The results of the redocking experiments showed that all three compounds had a good docking score in the binding pocket of pantothenate synthetase, as shown in Table 1.

**TABLE 1:** List of top three selected phytochemical compounds against pantothenate synthetase protein of *Mycobacterium tuberculosis* with their redocking result.

Compounds’ PubChem ID	Phytochemical name	VS result	Redocking result
5280805	**Rutin**	−11	−11
72307	**Sesamin**	−10.5	−10.5
6419835	**Catechin gallate**	−10.3	−10.3

**FIGURE 2:**
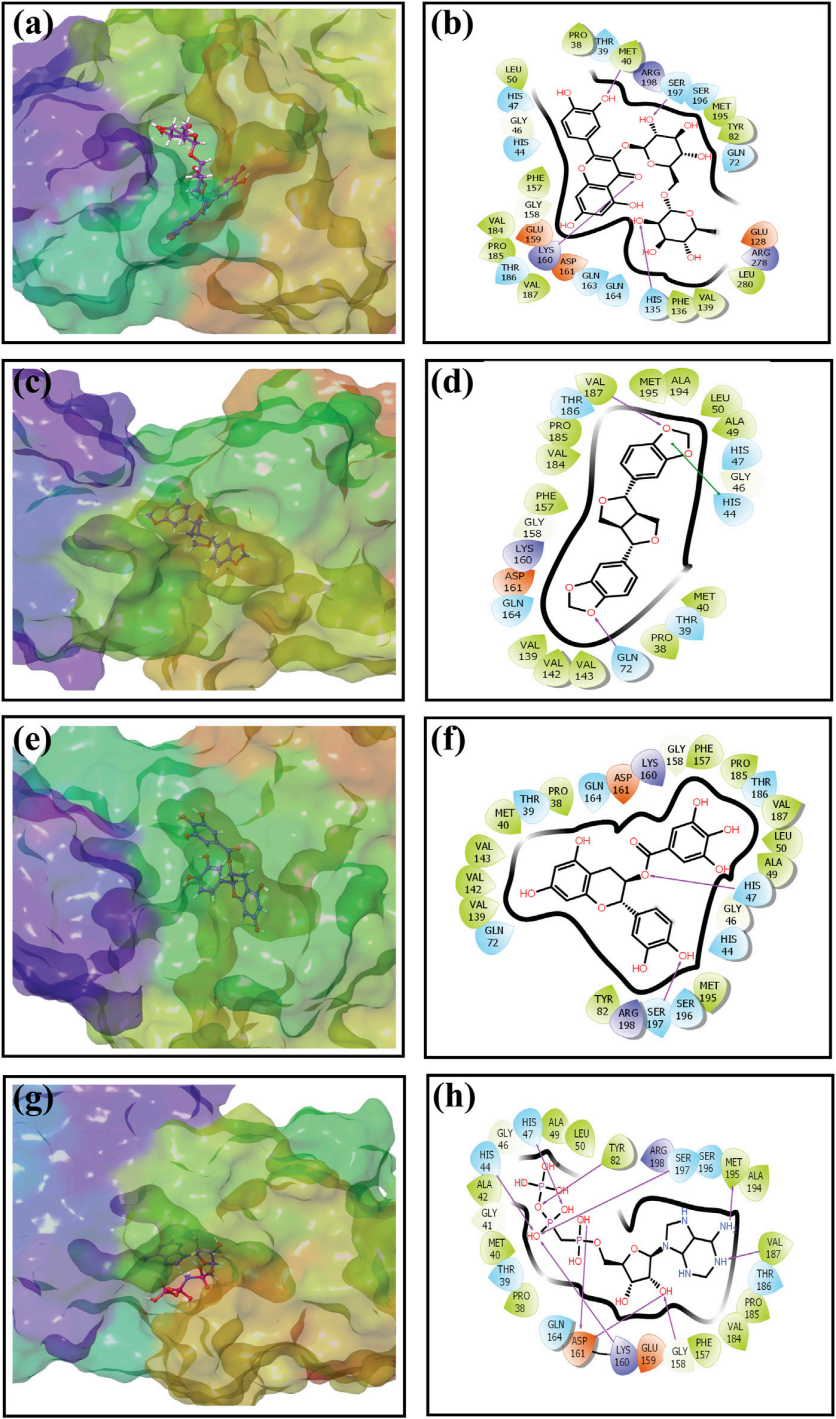
Three-dimensional and 2-dimensional docked complex poses of the selected phytochemical compounds and the reference compound, that is, **(A, B)** rutin, **(C, D)** sesamin, **(E, F)** catechin gallate, **(G, H)** and diphosphomethylphosphonic acid adenosylester, showing binding on the active site of the pantothenate synthetase protein. However, in 2-dimensional structures, H-bond formation (pink arrows), hydrophobic interaction (green), pi–pi stacking (green), polar residue (blue), negative residue (red), glycine (gray), and salt bridge (red and blue) interactions are logged for docked complexes of pantothenate synthetase protein with selected phytochemical compounds.

An intermolecular interaction study was carried out to understand the interaction profile between the each selected phytochemicals (rutin, sesamin, and catechin gallate) and the target protein (pantothenate synthetase), comparing the results with those obtained using the reference molecule, that is, diphosphomethylphosphonic acid adenosylester. The docked complex rutin–pantothenate synthetase showed binding energy −10 kcal/mol. This exhibited the involvement of four hydrogen bonds with residues Met^40^, His^135^, Lys^160^, and Ser^197^ and also showed other interactions such as hydrophobic interaction (Pro^38^, Met^40^, Leu^50^, Tyr^82^, Phe^136^, Val^139^, Phe^157^, Val^184^, Pro^185^, Val^187^, Met^195^, and Leu^280^ residues), polar interaction (Thr^39^, His^44^, His^47^, Gln^72^, His^135^, Gln^163^, Gln^164^, Thr^186^, Ser^196^, and Ser^197^ residues), negative (Glu^128^, Glu^159^, and Asp^161^ residues), positive (Lys^160^, Arg^198^, and Arg^278^ residues), and glycine (Gly^46^ and Gly^158^ residues) (Figures 2A, B). Similarly, the binding energy for sesamin–pantothenate synthetase was −10.5 kcal/mol, which showed two hydrogen bond formation (Gln^72^ and Val^187^ residues), and also exhibited involvement of additional interactions such as hydrophobic interactions (Pro^38^, Met^40^, Ala^49^, Leu^50^, Val^139^, Val^142^, Val^143^, Phe^157^, Val^184^, Pro^185^, Val^187^, Ala^194^, and Met^195^ residues), polar interaction (Thr^39^, His^44^, His^47^, Gln^72^, Gln^164^, and Thr^186^ residues), negative (Asp^161^ residue), positive (Lys^160^ residue), and glycine (Gly^46^ and Gly^158^ residues) (Figures 2C, D). Additionally, intermolecular interaction analysis of catechin gallate–pantothenate synthetase displayed that this docked complex was observed for two hydrogen bonds (His^47^ and Ser^197^ residues) and also exhibited involvement of additional interactions such as hydrophobic interactions (Pro^38^, Met^40^, Ala^49^, Leu^50^, Tyr^82^, Val^139^, Val^142^, Val^143^, Phe^157^, Pro^185^, Val^187^, and Met^195^ residues), polar interaction (Thr^39^, His^44^, His^47^, Gln^72^, Gln^164^, Thr^186^, Ser^196^, and Ser^197^ residues), negative (Asp^161^ residue), positive (Lys^160^ and Arg^198^ residues), and glycine (Gly^46^ and Gly^158^ residues) with a binding score (−10.3 kcal/mol) (Figures 2E, F). Moreover, the binding energy for the reference complex diphosphomethylphosphonic acid adenosylester–pantothenate synthetase was −10.4 kcal/mol, which is comparatively less than that of the docked complexes, that is, rutin–pantothenate synthetase and sesamin–pantothenate synthetase, but slightly higher than that of the catechin gallate–pantothenate synthetase docked complex. This reference complex showed the involvement of 10 hydrogen bonds (His^44^, His^47^, Tyr^82^, Gly^158^, Lys^160^, Asp^161^(2), Val^187^, Met^195^, and Ser^197^ residues) along with some extra interactions like hydrophobic interactions (Pro^38^, Met^40^, Ala^42^, Ala^49^, Leu^50^, Tyr^82^, Phe^157^, Val^184^, Pro^185^, Val^187^, Ala^194^, and Met^195^ residues), polar interaction (residues Thr^39^, His^44^, His^47^, Gln^164^, Thr^186^, Ser^196^, and Ser^197^), negative residual interaction (Glu^159^ and Asp^161^ residues), positive residual interaction (residues Lys^160^ and Arg^198^), and glycine (Gly^41^, Gly^46^, and Gly^158^ residues) (Figures 2G, H). It is also noted that the docked complex sesamin–pantothenate synthetase showed π–π stacking with His^44^ residue, and other than hydrogen bonding, all the three docked complexes presented approximately same hydrophobic interactions, polar interactions, negative residual interactions, positive residual interactions, and interactions with glycine, with only a few variations in specific amino acid residues. The similarity in interacting residues except the hydrogen bonding in these docked complexes suggested that the selected compounds interact with the pantothenate synthetase with almost the same binding affinity. In addition, the reference complex exhibited more hydrogen bonding than the selected docked complexes (Table 2). Therefore, by analyzing the result of redocking simulation and intermolecular interaction analysis, it is clear that the selected compounds were observed for good binding affinity.

**TABLE 2:** Molecular interaction profiling of top three selected phytochemical compounds and reference molecule, that is, (1) rutin, (2) sesamin, (3) catechin gallate, and (4) diphosphomethylphosphonic acid adenosylester, docked with pantothenate synthetase protein. Residues mentioned in the bold text are exactly similar to the residues displayed by the diphosphomethylphosphonic acid adenosylester.

Sr.no.	Drug	Binding energy	H-bond	Hydrophobic	π–π stacking	Polar	Negative	Positive	Glycine
1	**Rutin**	−11	Met^40^, His^135^, **Lys** ^**160**^, **Ser** ^**197**^	**Pro** ^**38**^, **Met** ^**40**^, **Leu** ^**50**^, **Tyr** ^**82**^, Phe^136^, Val^139^, **Phe** ^**157**^, **Val** ^**184**^, **Pro** ^**185**^, **Val** ^**187**^, **Met** ^**195**^, Leu^280^		**Thr** ^**39**^ **, His** ^**44**^ **, His** ^**47**^ **,** Gln^72^, His^135^, Gln^163^, **Gln** ^**164**^ **, Thr** ^**186**^ **, Ser** ^**196**^ **, Ser** ^**197**^	Glu^128^, **Glu** ^**159**^ **, Asp** ^**161**^	**Lys** ^**160**^ **, Arg** ^**198**^ **,** Arg^278^	**Gly** ^**46**^ **, Gly** ^**158**^
2	**Sesamin**	−10.5	Gln^72^, **Val** ^**187**^	**Pro** ^**38**^, **Met** ^**40**^, **Ala** ^**49**^, **Leu** ^**50**^, Val^139^, Val^142^, Val^143^, **Phe** ^**157**^, **Val** ^**184**^, **Pro** ^**185**^, **Val** ^**187**^, **Ala** ^**194**^, **Met** ^**195**^	His^44^	**Thr** ^**39**^ **, His** ^**44**^ **, His** ^**47**^, Gln^72^, **Gln** ^**164**^ **, Thr** ^**186**^	**Asp** ^**161**^	**Lys** ^**160**^	**Gly** ^**46**^ **, Gly** ^**158**^
3	**Catechin gallate**	−10.3	**His** ^**47**^, **Ser** ^**197**^	**Pro** ^**38**^, **Met** ^**40**^, **Ala** ^**49**^, **Leu** ^**50**^, **Tyr** ^**82**^, Val^139^, Val^142^, Val^143^, **Phe** ^**157**^, **Pro** ^**185**^, **Val** ^**187**^, **Met** ^**195**^		**Thr** ^**39**^ **, His** ^**44**^ **, His** ^**47**^ **,** Gln^72^, **Gln** ^**164**^ **, Thr** ^**186**^ **, Ser** ^**196**^ **, Ser** ^**197**^	**Asp** ^**161**^	**Lys** ^**160**^ **, Arg** ^**198**^	**Gly** ^**46**^ **, Gly** ^**158**^
4	**Reference inhibitor (diphosphomethylphosphonic acid adenosylester)**	−10.4	His^44^, His^47^, Tyr^82^, Gly^158^, Lys^160^, Asp^161^(2), Val^187^, Met^195^, Ser^197^	Pro^38^, Met^40^, Ala^42^, Ala^49^, Leu^50^, Tyr^82^, Phe^157^, Val^184^, Pro^185^, Val^187^, Ala^194^, Met^195^		Thr^39^, His^44^, His^47^, Gln^164^, Thr^186^, Ser^196^, Ser^197^	Glu^159^, Asp^161^	Lys^160^, Arg^198^	Gly^41^, Gly^46^, Gly^158^

### ADME profiling

**FIGURE 3:**
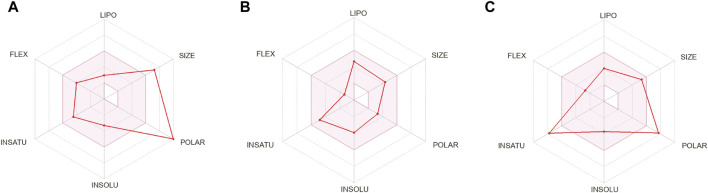
ADME profiling of the selected phytochemical compounds, that is, **(A)** rutin, **(B)** sesamin, and **(C)** catechin gallate.

The compounds that are suggested as drug candidates in the area of drug discovery must have strong biological activity coupled with minimal or negligible toxicity. Consequently, a set of crucial pharmacological measures, including pharmacokinetics and ADME parameters (absorption, distribution, metabolism, and excretion), have been proposed for the verification of each possible drug candidate. To comprehend and prevent pharmacokinetics-related failure of drug molecules in clinical trials, early evaluations of these parameters in the early stages of drug discovery are crucial (Hay et al., 2014). Thus, all of the screened phytochemical compounds, including rutin, sesamin, and catechin gallate (Figure 1), underwent assessments on the SwissADME for the evaluation of ADME properties in order to analyze the pharmacokinetic features and drug-likeness properties (Figure 3; Supplementary Table S5). Cytochromes (CY) CYP2D6, CYP1A2, CYP2C19, CYP2C9, CYP2D6, and CYP3A4 were found to be non-inhibitors of rutin and catechin gallate. These cytochromes are important for the metabolism of drugs and various xenobiotics, and their inhibition may result in decreased drug efficacy, drug activation, and drug metabolism. In contrast, sesamin inhibited several cytochromes. Additionally, rutin and catechin gallate have minimal absorption through the gastrointestinal tract and low blood–brain barrier (BBB) permeability. Sesamin, on the other hand, had high gastric absorption and BBB permeability. However, sesamin demonstrated zero violation for the Lipinski’s rule, whereas rutin and catechin gallate showed three and two violations, respectively (Supplementary Table S5). Rutin and catechin gallate also demonstrated violations of a number of additional drug-likeness standards, including Ghose, Veber, Egan, and Muegge, but sesamin exhibited no such violations. However, because cells can distinguish between natural molecules through their active transport system, natural compounds do not necessarily have to fulfill the requirements for drug-likeness (Lipinski, 2004; Macarron, 2006). Finally, the ADME study provided optimal therapeutic qualities for the chosen phytochemicals against the pantothenate synthetase protein. In addition, we have performed drug-likeness evaluation of all the ligands of screened library. The excel file of drug-likeness evaluation of these 239 compounds was added to the additional data of the Supplementary Material.

### MD simulation analysis

The stability and intermolecular interactions of docked complexes with respect to time are predicted using MD simulation, which sheds light on the dynamic behavior of a protein and the conformational alterations observed during ligand binding (Kumar et al., 2022). Throughout the complete 100 ns simulation, various parameters, including RMSD, RMSF, and protein–ligand contact profiling, were monitored to verify the stability of protein–ligand complexes. RMSD usually tracks the overall structural changes, providing the details on the conformational deviations and alterations in the docked protein–ligand complex. Conversely, the RMSF measures the local fluctuations and monitors deviation of each atom of the ligand and each residue of the protein in the presence of one another. Furthermore, the stability of the docked compound in the active site of the protein was analyzed through the protein–ligand contact profiling, which evaluates the interactions between the protein and the ligand throughout the simulation time.

Supplementary Figure S3 depicts the 3D structures of initial and last docked poses. Additionally, the 3D surface analysis of the final pose resulting from 100 ns MD simulation unveils significant conformational shifts in the pantothenate synthetase structure, when docked with phytochemicals, in comparison to its docking with the reference compound diphosphomethylphosphonic acid adenosylester (Supplementary Figure S1). This observation indicates the potential of docked phytochemicals to induce substantial alterations in the native conformational form of the pantothenate synthetase protein.

### RMSD and RMSF analyses

**FIGURE 4:**
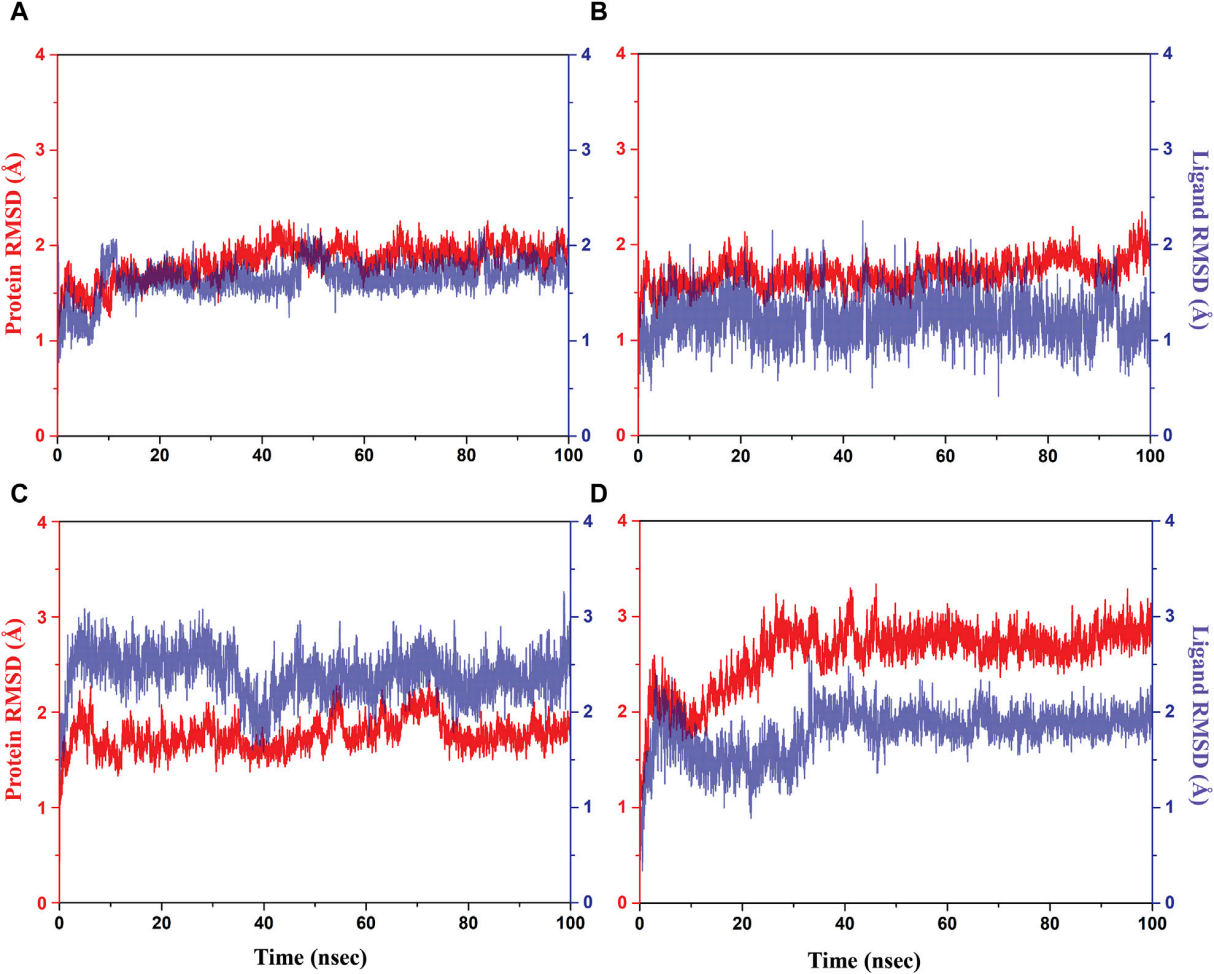
RMSD plots for the backbone atoms of the pantothenate synthetase protein and selected phytochemical compounds and the reference molecule, that is, **(A)** rutin, **(B)** sesamin, **(C)** catechin gallate, and **(D)** diphosphomethylphosphonic acid adenosylester fit on the selected target protein were extracted from 100 ns MD simulation trajectories of different docked complexes.

Initially, RMSD evaluation for both protein and ligand was documented for all docked complexes, using the initial pose as a reference frame (Figure 4). RMSD values for pantothenate synthetase displayed deviation of less than 2.5 Å in all the selected docked complexes, that is, rutin–pantothenate synthetase, sesamin–pantothenate synthetase, and catechin gallate–pantothenate synthetase docked complex. This observation signifies that the protein retained its conformation without getting any significant changes when interacting with the selected phytochemical compounds. On the other hand, all the three selected phytochemical compounds exhibited highly acceptable fluctuations (<3 Å), in which rutin and sesamin showed <2 Å RMSD, whereas catechin gallate displayed <3 Å RMSD. If we compare the RMSD of pantothenate synthetase in these selected complexes in contrast to the reference complex diphosphomethylphosphonic acid adenosylester–pantothenate synthetase, all the complexes including the reference complex showed highly acceptable mean deviation (<3 Å), but the protein in the reference docked complex exhibited slightly higher deviation than that in the selected phytochemical compound complexes. However, if we compare the RMSD of all the selected compounds and the reference compound in the docked complexes, we found that rutin displayed the same RMSD (2 Å), sesamin showed less RMSD (<2 Å), and catechin gallate exhibited greater RMSD (3 Å) than the reference compound. It is noteworthy that all the compounds and protein in all the docked complexes including the reference complex displayed highly acceptable RMSD. These data are supported by the RMSF analysis (Supplementary Figures S2, S3). Overall, RMSF values are essential for determining the local fluctuations between ligand molecules and protein chain. The local structural changes in the pantothenate synthetase protein and docked phytochemical compounds were identified as fluctuations caused in the phytochemical compound atoms and amino acid residues of the protein. Notably, the amino acid residues of pantothenate synthetase showed RMSF values within an acceptable range (<4 Å) for all the selected docked complexes and the reference complex except the amino terminal of the sesamin–pantothenate synthetase complex. In all the complexes, a peak was observed at amino acid residues present at 70 to 75 position, which is less than 3 Å in rutin and sesamin complexes and ∼4 Å in the catechin gallate complex, whereas this peak was >4 Å in the reference complex (Supplementary Figure S2). The RMSF plot of phytochemical compounds exhibited stability of phytochemical compounds with acceptable fluctuations (<2 Å) in atoms of phytochemicals. Atoms of sesamin and catechin gallate showed fluctuation up to 1 Å, whereas the atoms of rutin exhibited fluctuations (<1 Å) until 33 atoms then 35 and 36 atoms touch 2 Å fluctuations (Supplementary Figures S3A–C). On the other hand, the atoms of reference compound diphosphomethylphosphonic acid adenosylester also showed acceptable RMSF (2 Å) (Supplementary Figure S3D). For all three selected complexes, these RMSF and RMSD analyses supplied the necessary information to integrate the potential candidate into the binding site of pantothenate synthetase protein.

### Protein–ligand interaction mapping

**FIGURE 5:**
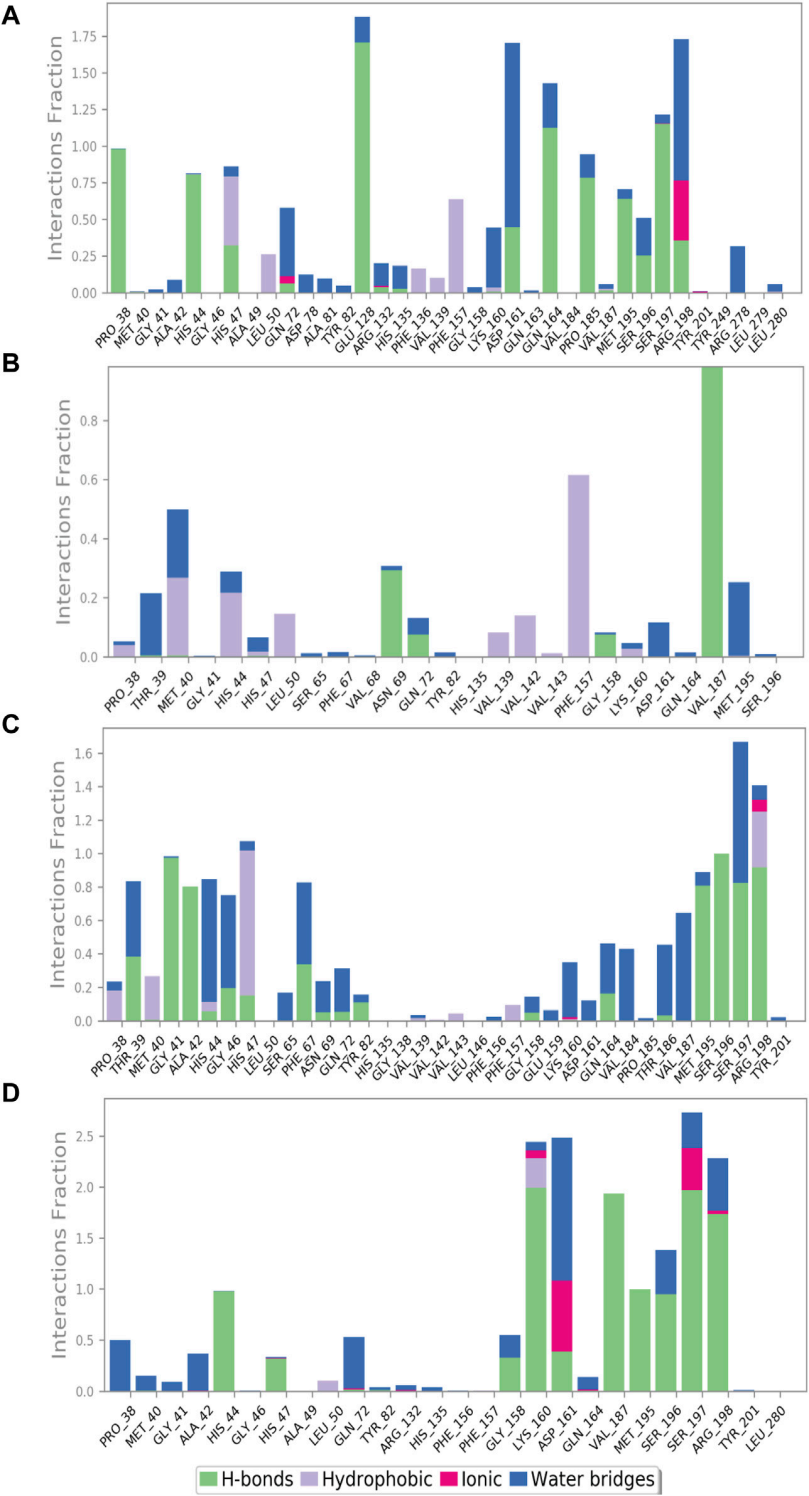
Protein–ligand interaction mapping for pantothenate synthetase protein docked with selected phytochemical compounds, that is, **(A)** rutin, **(B)** sesamin, **(C)** catechin gallate, and **(D)** diphosphomethylphosphonic acid adenosylester, extracted from 100 ns molecular dynamic simulations.

The protein–ligand interaction profile includes several interactions such as hydrogen bonding, hydrophobic contacts, ionic interactions, and formation of salt bridges. These parameters are crucial to comprehend how the protein and the ligand interactions change during the 100 ns MD simulation. We further investigated the atomic and intermolecular interactions that occurred during the 100 ns simulation of phytochemical substances with the active site residues of pantothenate synthetase (Figure 5).

During the whole simulation of the rutin–pantothenate synthetase docked complex, the predominant interactions were observed as hydrogen bonding and water bridges. Notably, the specific residues, namely, Pro^38^, Met^195^, Pro^185^ (noted for the formation of hydrophobic interaction in the docked complex), and His^44^ (exhibited polar interaction in the docked complex), showed the formation of hydrogen bonds more than 75% time of the simulation residues His^47^, Asp^161^, Ser^196^, and Arg^198^ (residues His^47^ and Ser^196^ displayed polar interaction, residue Asp^161^ observed for negative residual interaction, and residue Arg^198^ depicted positive residual interaction during the docking process) for greater than 30% of total MD simulation, and Glu^128^, Gln^163^, and Ser^197^ (residue Glu^128^ observed for negative residual interaction, whereas Gln^163^ and Ser^197^ displayed polar interaction during the respective protein–ligand docked complex) formed hydrogen bonds for over 100% of the whole simulation time. Residues His^47^ and Phe^157^ (residue His^47^ detected for polar interaction and Phe^157^ exhibited hydrophobic interaction during the docking process) displayed hydrophobic interaction for greater than 50% of the total simulation. Residues Arg^198^ and Asp^161^ (residue Arg^198^ depicted positive residual interaction and Asp^161^ exhibited negative residual interaction during the docking process) noted for the formation of water bridges for 100% and more than 100% of simulation, respectively, whereas Arg^198^ (noted for positive residual interaction in the same docked protein–ligand complex) also showed ionic interaction for over 25% of the whole simulation along with the formation of water bridges and hydrophobic interaction (Figure 5A).

Similarly, the docked complex sesamin–pantothenate synthetase was noted for the formation of hydrogen bonding with Val^187^ (depicted both hydrogen bond and hydrophobic interaction in the respective docked complex) and Asn^69^ (showed intermolecular interaction during simulation only) for more than 80% and 30% of the total period of simulation, respectively, whereas residue Phe^157^ (depicted for hydrophobic interaction during docking) involved in the hydrophobic interaction for more than 60% of the total interval of simulation. However, there are some residues which were involved in the formation of hydrogen bonding, hydrophobic interaction, and water bridges for less than 30% of the total simulation time (Figure 5B). In case of the docked complex, the catechin gallate–pantothenate synthetase complex displayed hydrogen bonding with Gly^41^, Ala^42^, Met^195^, Ser^197^, and Arg^198^ residues (residues Gly^41^ and Ala^42^ exhibited intermolecular interaction during simulation only, residue Met^195^ showed hydrophobic interaction, residue Ser^197^ depicted H-bonding and polar interaction, whereas residue Arg^198^ displayed positive residual interaction in the docked complex) for over than 80%, residue Ser^196^ (showed polar interaction in the docked complex) for 100%, and some residues less than 40% of the simulation time. Residue Ser^197^ (noted for both hydrogen bond formation and polar interaction during the docking process) and residue His^44^ (polar interaction exhibited by the same residue in the respective protein–ligand docked complex) were noted for water bridge formation for 80% and 70% of MD simulation, respectively. However, there were some residues which were observed for relatively shorter period of the MDS time. Additionally, Arg^198^ and Lys^160^ (both noted for positive interactions only in the respective docked complex) were involved in ionic interaction for a very short period of the total simulation time (Figure 5C). However, protein–ligand interaction mapping of the reference complex, that is, diphosphomethylphosphonic acid adenosylester–pantothenate synthetase, was comparatively analyzed, where residues His^44^, Met^195^, and Ser^196^ (residue His^44^ exhibited H-bonding and polar interaction, residue Met^195^ showed H-bonding and hydrophobic interaction, and residue Ser^196^ exhibited polar interaction during the docking process) formed hydrogen bonds for 100%, and residues Lys^160^, Val^187^, Ser^197^ (these three residues presented H-bonding with positive, hydrophobic, and polar interactions in the docked complex, respectively), and Arg^198^ (showed positive interaction only in the protein–ligand docked complex) for 100% of the total simulation interval. However, residues Lys^160^ (formed H-bonds and positive residual interaction in the same docked complex) and Leu^50^ (detected for hydrophobic interaction only during docking) were observed for hydrophobic interaction for a short period of MDS. However, Asp^161^ (presented H-bond formation and negative residual interaction in the same docked complex) was detected for the formation of water bridges for more than 100% of the simulation interval, and additionally, some residues also showed interaction for water bridge formation for less than 50% of the MDS time. It is noteworthy that Asp^161^ and Ser^197^ exhibited ionic interaction along with hydrogen bonding and hydrophobic interaction (Figure 5D). Selected phytochemical compounds can be used as effective inhibitors of the target pantothenate synthetase with reference to the study of these complex analyses due to the formation of potent hydrogen bonds, water bridges, and engagement in hydrophobic interactions within the active pocket of the target protein pantothenate synthetase.

### Ligand–protein contacts

**FIGURE 6:**
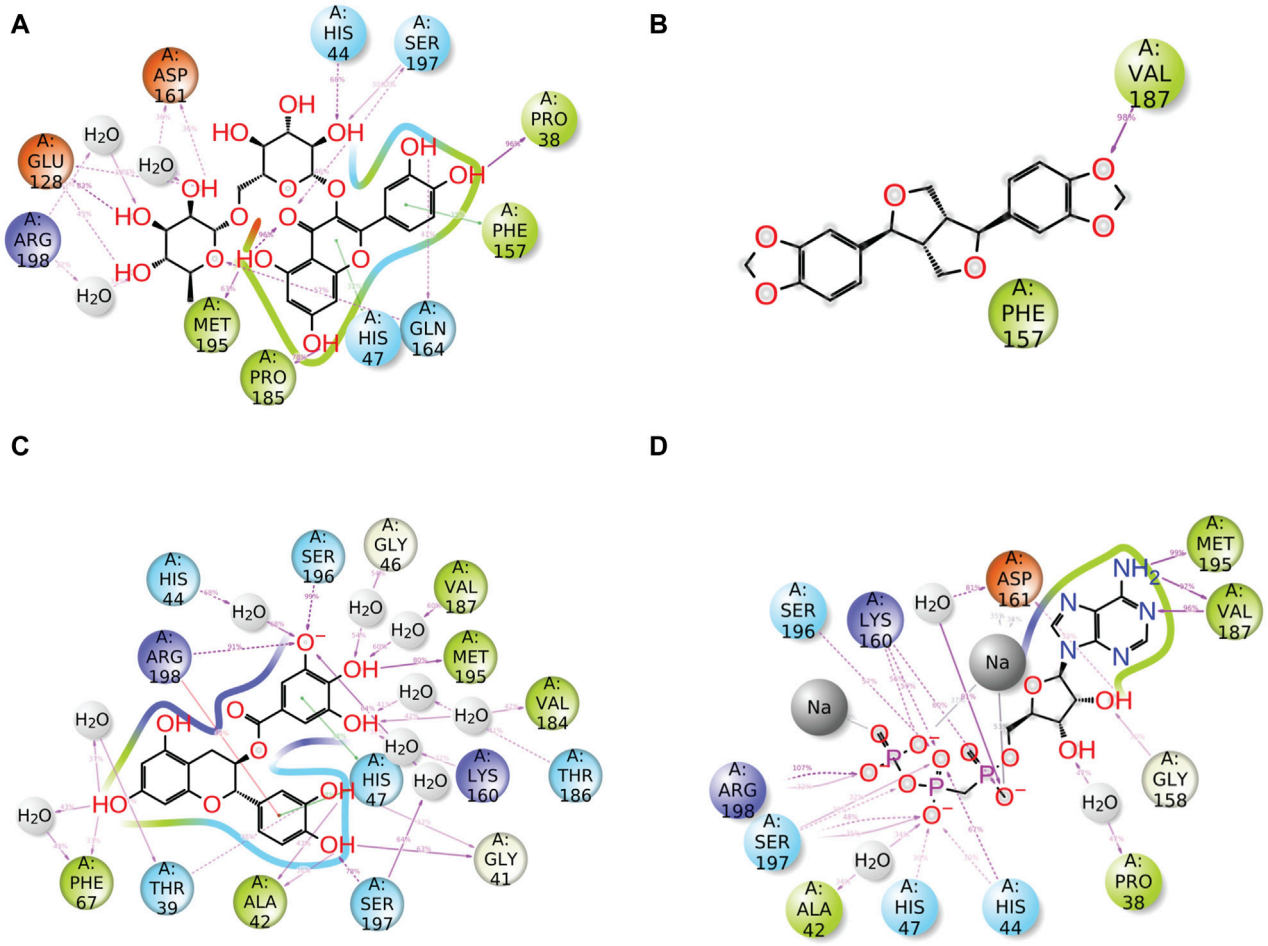
Detailed schematic representation of atomic interaction of ligand of phytochemical compounds and the reference molecule, that is, **(A)** rutin, **(B)** sesamin, **(C)** catechin gallate, and **(D)** diphosphomethylphosphonic acid adenosylester, docked with pantothenate synthetase protein. On the chosen trajectory (0.00–100.01 nsec), interactions that occur more than 30.0% of the simulation period are displayed.

Furthermore, after the analysis of docked complexes, it was observed that the ligands interact with specific residues of protein for a significant portion (over 30%) of the simulation time. This schematic representation provides valuable insights into the dynamic behavior of the ligand–protein interaction. When compared to the reference complex, it was observed that all of the chosen natural compounds significantly contributed to the dynamic stability of the pantothenate synthetase protein through hydrogen bonding, pi–pi stacking, pi–cation interaction, and hydrophobic and negative contact (Figure 6). Compound rutin exhibited 14 H-bonds with 9 residues (Pro^38^, Ser^197^ (2), His^44^, Asp^161^ (2), Glu^128^ (2), Arg^198^ (2), Met^195^, Pro^185^, and Gln^164^ (2)); among them, residues Asp^161^ and Arg^198^ (2) exhibited three water-mediated H-bonds with 36%, 32%, and 45% of the total frames produced during MD simulation. However, the pi–pi stacking interaction was presented by both residues Phe^157^ and His^47^ (Figure 6A). Compound sesamin showed only one H-bond by residue Val^187^ of the docked protein with 98% of the total frames of MDS (Figure 6B). Compound catechin gallate in the docked complex displayed 19 H-bonds with 14 residues (Gly^41^ (2), Ser^197^ (2), Ala^42^ (2), Thr^39^ (2), Phe^67^ (2), Arg^198^, His^44^, Ser^196^, Gly^46^, Val1^87^, Met^195^, Val^184^, Thr^186^, and Lys^160^), of which 9 residues were noted for the formation of water-mediated H-bond with more than 32% of the total frames produced from MD simulation. Residue Arg^198^ was observed for the formation of H-bonding and pi–cation interaction with 91% and 33% of the total frames of the whole simulation interval, respectively (Figure 6C). On the other hand, the reference compound, that is, diphosphomethylphosphonic acid adenosylester, presented 20 H-bonds by 12 residues; among them, only 3 residues exhibited water-mediated H-bonding (Figure 6D). As a result, when evaluating the stability of the docked complexes using MD simulations and protein–ligand interaction profiling, we can say that these selected docked complexes appear to be stable in the binding site of pantothenate synthetase (Figure 6).

### Radius of gyration

**FIGURE 7:**
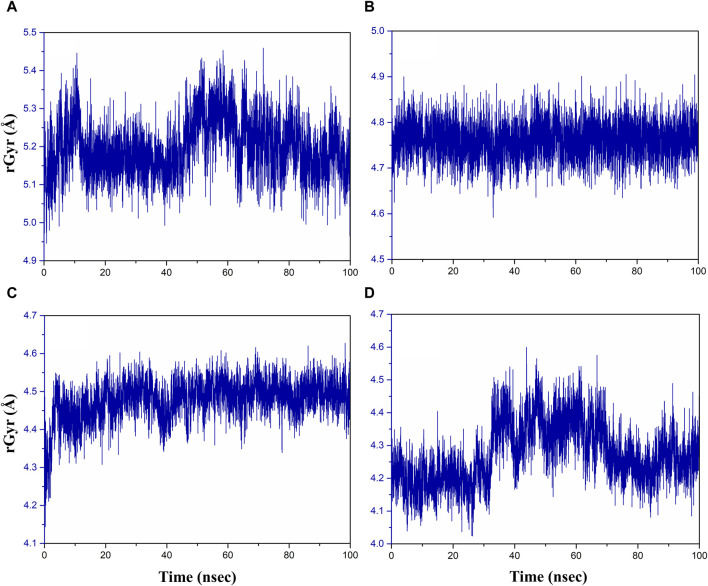
Radius of gyration plot of the **(A)** rutin–pantothenate synthetase, **(B)** sesamin–pantothenate synthetase, **(C)** catechin gallate–pantothenate synthetase, and **(D)** diphosphomethylphosphonic acid adenosylester–pantothenate synthetase.

The radius of gyration (rGyr) is used to analyze the overall compactness and conformational changes of the protein during the simulation (Rampogu et al., 2022). It provides insight into the size of protein and how it fluctuates over time. rGyr is calculated based on the positions of the atoms in the protein. An increase in rGyr may indicate protein unfolding or expansion, whereas a decrease may indicate compaction or folding (Thirumalai et al., 2019). The results of rGyr are shown in Figure 7, with average rGyr values ranging from 4.47 to 5.19 Å for all the phytochemical complexes, whereas the reference complex exhibited the rGyr value of 4.27 Å.

Figure 7 demonstrates that over the course of the simulation, the catechin gallate–pantothenate synthetase system displayed a lower rGyr with an average of 4.47 Å, whereas sesamin-pantothenate synthetase (4.76 Å) and rutin–pantothenate synthetase (5.19 Å) displayed a relatively higher rGyr. These findings imply that in contrast to the other compounds, catechin gallate exerts conformational stability and compactness inside the pantothenate synthetase protein.

#### Molecular interactions of lead molecules vs. control molecule

Detailed mechanistic studies are essential to unravel how compounds interact with pantothenate synthetase at the molecular level, and we have analyzed the crystal structures of pantothenate synthetase complexed with inhibitors (PDB: 1N2H, 1N2E and 1N2G) to understand the binding mode and key interactions with pantothenate synthetase to gain insight about their binding stability and inhibition (Wang and Eisenberg, 2003). The simulation studies on the crystal complex of pantothenate synthetase and a known inhibitor for comparative analysis of interactions dynamics were also performed. Furthermore, the mode of binding of docked ligands with crystal conformation of known inhibitors was also compared. Interactions between newly identified potential inhibitors and target proteins are similar to interactions between control inhibitors and target proteins. Moreover, the interaction profile during MD simulation was also very similar (Supplementary Figures S4, 5). Additional interactions were observed in the modeled complex with new molecules, and they were found to be intact during simulations. These observations suggest that newly identified molecules would inhibit the target enzyme with higher potency.

Moreover, blind docking was performed to find out the most likely binding site in the protein for the three newly identified ligands. We found that ligand docked into the active site and not into any other pocket. This also indicates that this hit would compete with the substrate; hence, it can act as a competitive inhibitor (Supplementary Figure S4).

In the crystal complex, the control inhibitor partially occupied the pantonate binding pocket while being bound to the primary binding site. The purine base moiety of the ligand interacted with active site residues Val^187^ and Met^195^, forming hydrogen bonds (Supplementary Figures S3A, B). The phosphate group oxygen formed a hydrogen bond with the main chain of Met^40^, which is essential for pantonate adoption, alongside Gln^164^, Gln^72^, and a water molecule, contributing to ligand stability as observed in crystal complexes (Supplementary Figure S3A).

The purine moiety of the control ligand interacted with Val^187^, Leu^50^, Phe^157^, Pro^185^, and Met^195^ through van der Waals contacts. This pocket, occupied by the phenyl ring of catechin and rutin inhibitors, featured the catechol moiety of the lead molecule (Supplementary Figure S3B). During simulations, the main chain of Val^187^ and the amine group of purine formed additional hydrogen bonds similar to the free amino group control ligand. This extended moiety interacted with non-polar side chains, predominantly forming van der Waals interactions.

In the catechin complex, Arg^198^ formed a salt bridge with an oxide ion and participated in seven hydrogen bonds and a pi–pi interaction. Additional residues like Ala^42^ and Ser^196^ contributed to hydrogen bond formation, with common van der Waals interactions among residues (Supplementary Figure S3D). Similarly, the sesamin complex retained interactions from the docked conformations without substantial changes (Supplementary Figure S3E).

Compared to the control molecule, rutin and catechin displayed superior interactions due to the presence of critical residues like His^47^, Gln^72^, Glu^128^, and Phe^157^ (Supplementary Figures S3C, D). These additional interactions enhanced the stability of the lead complexes, making rutin and catechin promising lead candidates. Modeling studies suggest rutin and catechol’s potential to bind to PS and inhibit it, with increased binding affinity due to additional interactions compared to control inhibitors. Compared to the control molecule, rutin and catechin exhibit superior interactions due to the presence of critical residues like His^47^, Gln^72^, Glu^128^, and Phe^157^. These additional interactions enhance the stability of the lead complexes, making rutin and catechin superior to the control molecule as potential lead candidates.

Overall, the observed interactions in molecular modeling studies highlight the potential of rutin and catechol as inhibitors, underscoring their promise for further investigation in inhibiting pantothenate synthetase and related targets.

### MM/GBSA

**FIGURE 8:**
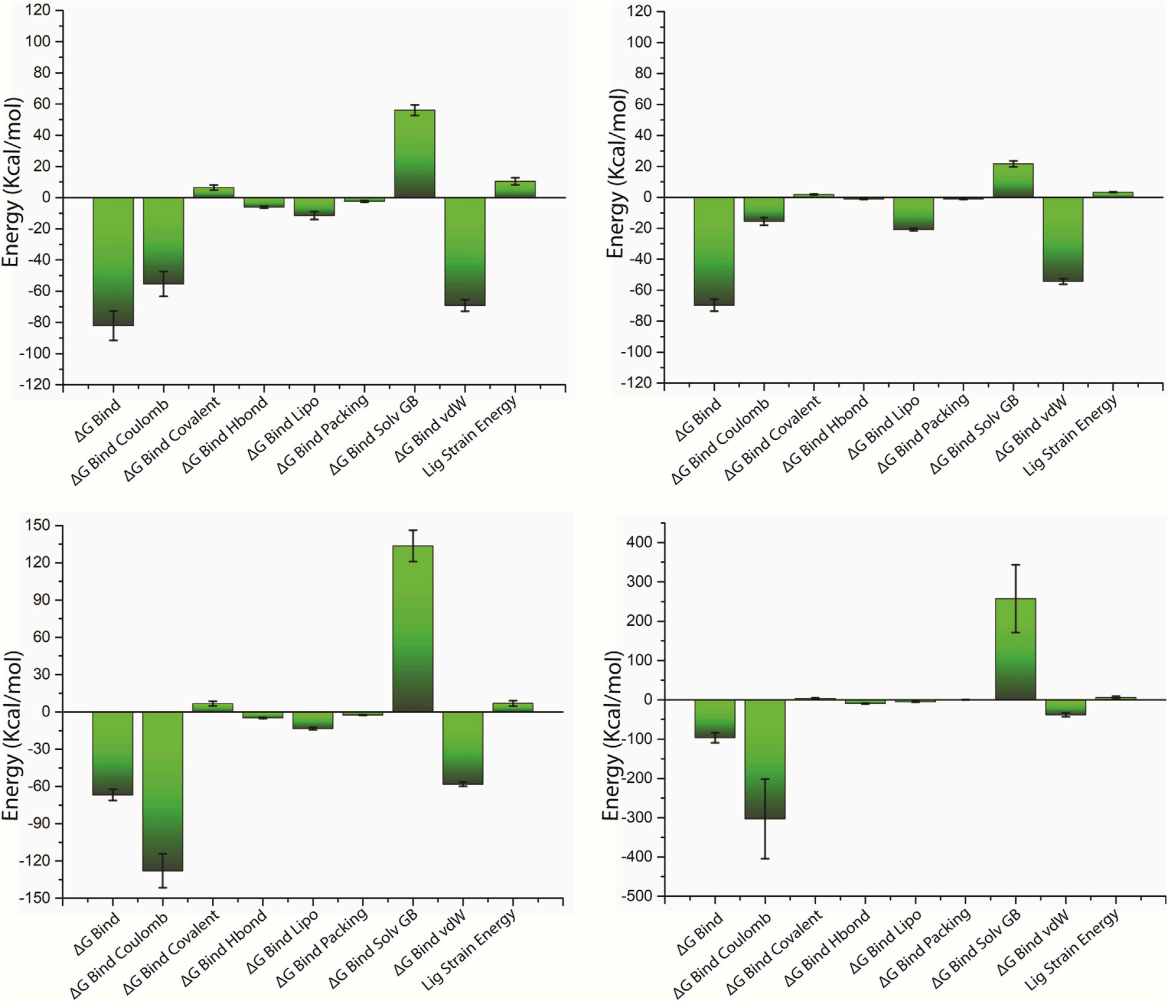
Calculated net binding free energy and energy components values for the pantothenate synthetase protein complex with selected phytochemical compounds, that is, **(A)** rutin, **(B)** sesamin, **(C)** catechin gallate, and **(D)** diphosphomethylphosphonic acid adenosylester; snapshots extracted from last 10 ns MD simulation trajectory.

The net binding free energy of the retrieved poses from the last 10 ns MD simulation trajectory of the respective docked complexes was calculated using the MM/GBSA method. Furthermore, energy dissociation components were computed in order to predict their contribution to the overall stability of the identified possible phytochemical docked with the target protein, that is, pantothenate synthetase. When performing a comparative analysis with the corresponding reference complex, the evaluation of net binding free energy for the screened phytochemicals docked with the pantothenate synthetase revealed considerable energy values. Interestingly, in comparison to other discovered phytochemicals, rutin docked with pantothenate synthetase had the largest negative free binding energy (Supplementary Table S6; Figure 8).

On comparing net binding free energy of the reference diphosphomethylphosphonic acid adenosylester–pantothenate synthetase complex, that is , −96.44 ± 12.82 kcal/mol, all of the complexes of phytochemicals docked with the pantothenate synthetase exhibited comparatively lower binding free energy but in the acceptable range (Figure 8D; Supplementary Table S6). The docked complex rutin–pantothenate synthetase showed higher binding free energy (−82.24±9.35 kcal/mol), whereas sesamin–pantothenate synthetase and catechin gallate–pantothenate synthetase exhibited lower but still considerable binding free energy values (−69.78 ± 3.88 kcal/mol and −66.83 ± 4.5 kcal/mol, respectively). Furthermore, the dissociation energy components of all docked complexes were estimated, with ∆G Bind vdW and ∆G Bind Lipo contributing to the stability of the complex and the instability was determined by ∆G Bind Solv GB in the respective complexes (Figure 8 and Supplementary Table S6). Notably, ∆G Bind vdW and ∆G Bind Lipo of the three selected complexes have a larger value than those of the reference complex; however, ∆G Bind Solv GB of the three selected complexes has a lower value than that of the reference complex. These findings revealed that rutin had the highest affinity and stability for pantothenate synthetase, followed by sesamin and catechin gallate. As a result, MM/GBSA analysis estimated by the net binding free energy calculation assists the three selected common phytochemicals to be possible pantothenate synthetase protein inhibitors.

Along with MM/GBSA, we also incorporated the time-dependent interaction profile during simulation to evaluate the stability of protein–ligand complexes in the binding pocket (Supplementary Figure S6). Greenidge et al. (2014) found that MM/GBSA displayed stronger correlation with experimental binding affinity than empirical scoring functions used by docking programs (Greenidge et al., 2014). There are also several intrinsic limitations of MM/GBSA for calculating binding affinity.

### Principal component analysis

**FIGURE 9:**
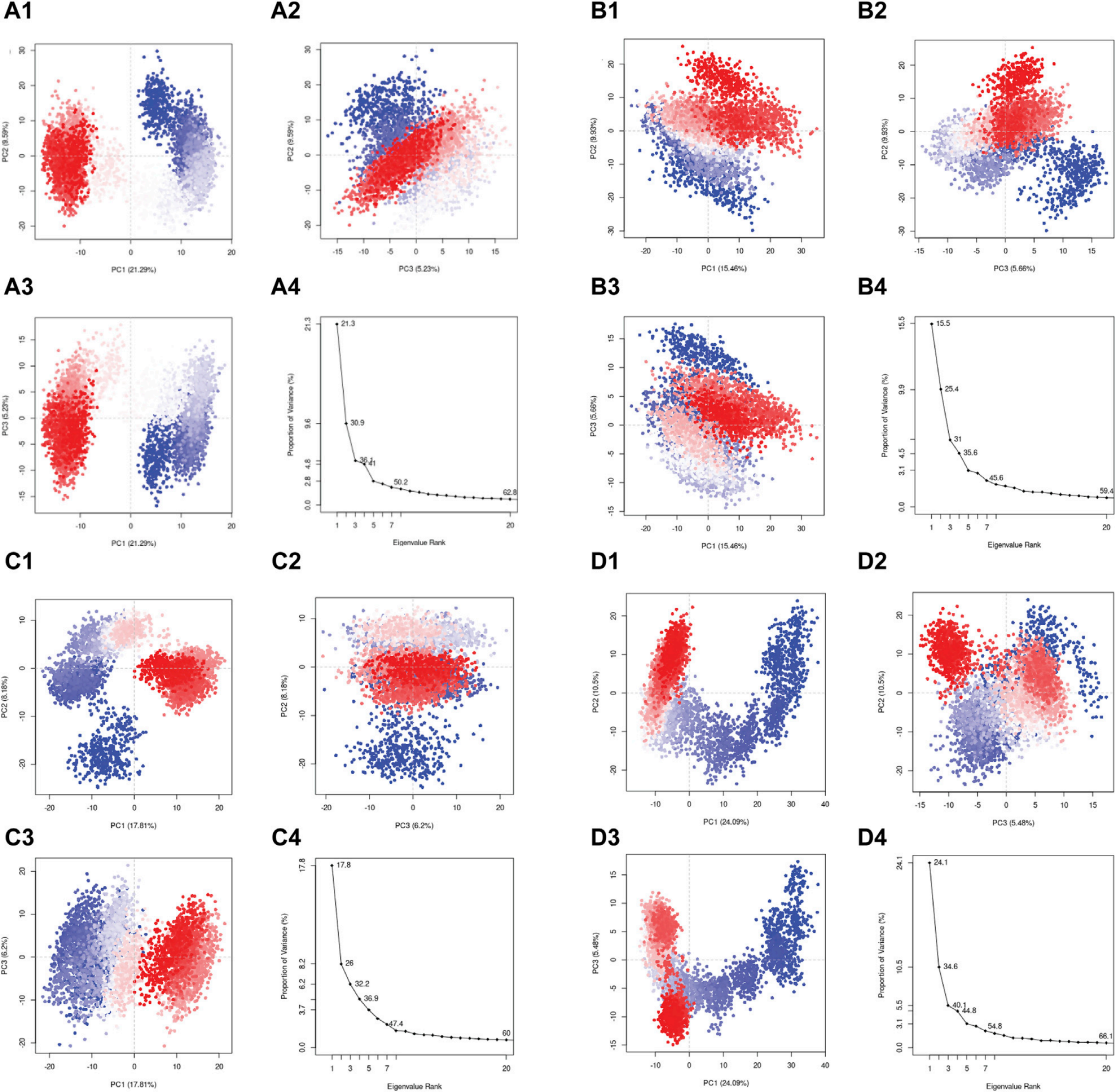
Principal component analysis for the molecular dynamic simulation trajectories of pantothenate synthetase docked with **(A1–4)** rutin, **(B1–4)** sesamin, **(C1–4)** catechin gallate, and **(D1–4)** diphosphomethylphosphonic acid adenosylester. The percentage of total mean square displacement of residue positional variations recorded in each dimension is categorized by equivalent eigenvalue (PCs). The persistent color scale from blue to white to red directs the periodic jumps between the protein conformers duration the 100 ns simulation interval.

In the current study, the conformational mobility of the protein after binding to the selected compound was assessed using the PCA, and it was also calculated for the reference complex to facilitate the comparison study. During the simulation, a protein molecule moved around in the system in multiple dimensions. PCA is used to reduce the dimensions into basic fundamental components. The initial critical first three elements (eigen vectors) for each complex were investigated in the present study. These were supposed to be the statistically significant protein structural movements acquired from simulation. Similarly, Figures 9D1–4 depict the top three major components of the reference complex. Figures 9A–C demonstrate the PCA of the top three hits’ complexes and the reference. In the first three principal components, rutin, sesamin, and catechin gallate have 36.1%, 31%, and 32.2% movement coverage, respectively. Figure 9 shows the plots of the PCA with multiple data points, each representing the protein’s conformation. The plots show a color gradient (blue–red) from the beginning to the end of the simulation. In this case, sesamin demonstrated a lesser conformational variation in the protein structure. Additionally, the reference compound had movement coverage of 40.1% in its three initial principal components (PCs). In addition, this had more overlap in the PC2 and PC3 plots, whereas the dispersion demonstrated by PC1 and PC2, PC1, and PC3 plots exhibited a higher relative variation shown in the plots in Figure 9. Nonetheless, the PCA of all three complexes indicates that the initial three PCs are sufficient to explain a large proportion of the overall variance in the data, but the rutin complex exhibited the percent of variance which is slightly similar to the first three PCs of the control compound. This shows that the initial three PCs in the rutin complex are more essential to explain the total variance, as shown in Figure 9. The rutin complex exhibited a cluster which is red in color with lower variance, whereas the cluster with blue color had larger variance. The value of PC1 is generally thought to be larger than that of PC2 and PC3, and it exhibits the greatest variations in protein conformations during simulation. However, in the PC2 and PC3 plots, other compounds’ complexes overlap more. When focusing on all three PCAs, Figures 9A1–4, D1–4 show that rutin–pantothenate synthetase is the most similar to the reference ligand.

To date, there were so many chemically synthesized drugs discovered against pantothenate synthetase in *Mtb*. However, a notable distinction arises when considering compounds of natural origin. Only natural compounds such as seaweed metabolites (fucoidan and kappa carrageen) and neem compound (nimocinolide) were identified as potential drugs targeting the active site of pantothenate synthetase. Expanding the scope of this study, we turned our attention to phytochemicals derived from plants which are known for their antibacterial properties.

In 2023, Rajan et al., conducted a study in which two seaweed metabolites, that is, fucoidan and kappa carrageen, were identified. These metabolites showed binding energies of −5.57 kcal/mol and −2.73 kcal/mol, respectively (Arokia Rajan et al., 2023). In addition, Mahanta et al. (2021) used neem-derived compounds and identified nimocinolide with a binding affinity of −8.4 kcal/mol as a potential lead compound for the development of a new drug against *Mtb* pantothenate synthetase (Mahanta et al., 2021). Devi et al. (2015) screened a commercial database, Asinex, containing 500,000 compounds and identified lead compound 10 with a binding affinity of −8.4 kcal/mol (Devi et al., 2015). Furthermore, Hassan et al. (2020) synthesized various hybrid molecules by incorporating pyrazine scaffold and anti-mycobacterial moieties and screened 31 compounds for activity against *Mtb* using the MABA assay. Out of them, six compounds (8a, 8b, 8c, 8d, 14b, and 18) displayed significant activity with MIC values ≤6.25 μg/mL. Furthermore, ADMET and drug-like property analysis for those derivatives have shown promising candidates (Hassan et al., 2020). However, this work has demonstrated three phytochemicals, namely, rutin, sesamin, and catechin gallate, with strong binding affinity against pantothenate synthetase (binding energy ranging from −11 to −10.3 kcal/mol) as compared to fucoidan, kappa carrageen, nimocinolide, and other lead compounds (Devi et al., 2015; Mahanta et al., 2021; Arokia Rajan et al., 2023). This revealed that three selected compounds of our study hold a prominent position, making them potential candidates for further validation studies.

## Conclusion

Pantothenate synthetase is a bacterial protein that is a potential drug target for the development of anti-tubercular treatments due to its critical function in a number of *Mtb* cellular functions and the absence of relevant homologs in humans. Beyond conventional synthetic medications, the field of pharmacology has evolved, with a growing emphasis on using the therapeutic potential of phytochemicals originating from plants. Thus, this work used molecular docking, drug-likeness, MD simulation, and binding free energy calculations to assess the potential and therapeutic effectiveness of phytochemicals from various plants against pantothenate synthetase. Notably, out of 239 plant-based compounds, the top three phytochemicals, that is, rutin, sesamin, and catechin gallate, were chosen as bispecific inhibitors of pantothenate synthetase, which exhibit high binding affinity and dynamic stability. In addition, these selected compounds exhibited superior binding affinity for pantothenate synthetase, with binding energies ranging from −11 to −10.3 kcal/mol. This indicates that it has a higher binding affinity than the previously studied compounds, namely, fucoidan, kappa–carrageenan, and nimocinolide, which have been investigated for their interaction with pantothenate synthetase. Overall, the current research suggested that predicted phytochemicals may be used as drugs for *Mtb* treatment. However, further precise experimental validation of these compounds under *in vitro* and *in vivo* is needed to assess their potential as an inhibitor for pantothenate synthetase to inhibit *Mtb*.

## Data Availability

The original contributions presented in the study are included in the article/Supplementary Material. Further inquiries can be directed to the corresponding authors.
