# Supplementary material for: Unearthing phytochemicals as natural inhibitors for pantothenate synthetase in Mycobacterium tuberculosis: A computational approach
Source: Front Pharmacol. 2024 Jul 29;15:1403900. doi: 10.3389/fphar.2024.1403900 (PMC11317409; doi:10.3389/fphar.2024.1403900)
Supplement: Supplementary file 1 [file DataSheet1.docx]

**Table S1.** Phytochemical compounds based on paper.

| Chemical classes of Phytochemicals | Phytochemical compounds | PubChem ID |
| --- | --- | --- |
| Alkaloids | morphine | 5288826 |
|  | strychnine | 441071 |
|  | emetine | 10219 |
|  | brucine | 442021 |
|  | piperine | 638024 |
|  | caffeine | 2519 |
|  | quinine | 3034034 |
|  | colchicine | 6167 |
|  | coniine | 9985 |
|  | ephedrine | 9294 |
|  | vincamine | 15376 |
|  | reserpine | 5770 |
|  | quinidine | 441074 |
|  | cocaine | 446220 |
|  | nicotine | 89594 |
|  | sanguinarine | 5154 |
|  | Berberine | 2353 |
|  | tomatidine | 65576 |
|  | 6-gingerol | 442793 |
|  | cinchonidine | 101744 |
|  | cinchonine | 90454 |
|  |  |  |
| Sulfur-containing phytochemicals | allicin | 65036 |
|  | ajoene | 5386591 |
|  | sulforaphane | 5350 |
|  | berteroin | 206037 |
|  | hirsutin | 9794659 |
|  | phenethyl-isothiocyanate | 16741 |
|  | Alyssin | 206035 |
|  | erucin | 78160 |
|  | Allyl-isothiocyanate | 5971 |
|  | Benzyl-isothiocyanate | 2346 |
|  | 2-phenylethyl-isothiocyanate | 16741 |
|  | 2-(4-hydroxyphenyl) ethyl-isothiocyanate | 10442282 |
|  | Phenyl-isothiocyanate | 7673 |
|  |  |  |
| Terpenoids | thymol | 6989 |
|  | carvacrol | 10364 |
|  | eugenol | 3314 |
|  | trans-cinnamaldehyde | 637511 |
|  | Beta-resorcylic acid | 1491 |
|  | vanillin | 1183 |
|  | Limonene | 22311 |
|  | Salvipisone | 821448 |
|  | aethiopinone | 157301 |
|  | Farnesol | 445070 |
|  | Oleanolic acid | 10494 |
|  | Ergosterol peroxide | 5351516 |
|  | bonianic acid A | 53356107 |
|  | bonianic acid B | 53356108 |
|  | Ursolic acid | 64945 |
|  | Alpha-amyrin | 73170 |
|  | betulinic acid | 64971 |
|  | betulinaldehyde | 99615 |
|  | 3-O-acetyluncaric acid | 53356215 |
|  | alpha-carotene | 6419725 |
|  | beta-carotene | 5280489 |
|  | beta-cryptoxanthin | 5281235 |
|  | lutein | 5281243 |
|  | zeaxanthin | 5280899 |
|  | rubixanthin | 5281252 |
|  |  |  |
| Polyphenols | galangin | 5281616 |
|  | kaempferol | 5280863 |
|  | quercetin | 5280343 |
|  | myrecetin |  |
|  | Luteolin | 5280445 |
|  | Chrysin | 5281607 |
|  | Naringenin | 932 |
|  | Hesperetin | 72281 |
|  | Malvidin | 159287 |
|  | Demethyltexasin | 5284649 |
|  | hydroxydaidzein | 5280520 |
|  | biochanin A | 5280373 |
|  | demethylretusin |  |
|  | genistein | 5280961 |
|  | Daidzein | 5281708 |
|  | Boropinic acid | 10682896 |
|  | Gallic acid | 370 |
|  | Resveratrol | 445154 |
|  | Lacinartin | 5324625 |
|  | Coumarins | 54678486 |
|  | Dihydroxybergamotin | 101046549 |
|  | bergamottin | 5471349 |
|  | 1,2,6-tri-O-galloyl-beta-d-glucopyranose | 124024 |
|  | Proanthocyanidins | 107876 |
|  | apigenin | 5280443 |
|  | catechin | 9064 |
|  | epicatechin | 72276 |
|  | epigallocatechin | 72277 |
|  | epicatechin gallate | 107905 |
|  | epigallocatechin gallate | 65064 |
|  | eriodictyol | 440735 |
|  | cyanidin | 128861 |
|  | pelargonidin | 440832 |
|  | peonidin | 441773 |
|  | glycitein | 5317750 |
|  | daidzin | 107971 |
|  | coumaric acid | 637542 |
|  | ferulic acid | 445858 |
|  | chlorogenic acid | 1794427 |
|  | petunidin | 441774 |
|  | Auraptene | 1550607 |

**Table S2.** Extra phytochemical compounds added to the phytochemical compounds based on paper.

|  | Plant Name | Constituents/ Phytochemicals | PubChem ID | Phytochemical Class |
| --- | --- | --- | --- | --- |
| Spices | Allium sativum | Alliin | 121922 | Organosulfurs |
|  |  | Diallyl disulfide | 16590 | Organosulfurs |
|  |  | Diallyl sulfide | 11617 | Organosulfurs |
|  |  | Diallyl trisulfide | 16315 | Organosulfurs |
|  | Cinnamomum cassia | Vanillic acid | 8468 | Polyphenols |
|  | Cinnamomum tamala | camphene | 6616 | Terpenoids |
|  |  | Caryophyllene oxide | 1742210 | Terpenoids |
|  |  | Humulene oxide | 6324 | Terpenoids |
|  |  | Methyl eugenol | 7127 | Terpenoids |
|  |  | P-CYMENE | 7463 | Terpenoids |
|  |  | phenetol | 7674 | Polyphenols |
|  |  | spathulenol | 92231 | Terpenoids |
|  |  | Valencene | 9855795 | Terpenoids |
|  |  | alpha-Humulene | 5281520 | Terpenoids |
|  |  | ALPHA-PINENE | 6654 | Terpenoids |
|  |  | BETA-PHELLANDRENE | 11142 | Terpenoids |
|  |  | BETA-PINENE | 14896 | Terpenoids |
|  | Cinnamomum zeylenicum | alpha-Bergamotene | 86608 | Terpenoids |
|  |  | ALPHA-COPAENE | 19725 | Terpenoids |
|  |  | BETA-CARYOPHYLLENE | 5281515 | Terpenoids |
|  |  | linalool | 6549 | Terpenoids |
|  | Cuminum cyminum | Cuminaldehyde | 326 | Terpenoids |
|  |  | Cuminoside A | 1012522065 | Polyphenols |
|  |  | Cuminoside B | 1012522066 | Polyphenols |
|  |  | Thymoquinone | 10281 | Terpenoids |
|  | Foeniculum vulgare | 5-Methoxypsoralen | 2355 | Polyphenols |
|  |  | Acacetin | 5280442 | Polyphenols |
|  |  | alpha-Phellandrene | 7460 | Terpenoids |
|  |  | Estragol | 8815 | Polyphenols |
|  |  | Fenchone | 14525 | Terpenoids |
|  |  | Isorhamnetin | 5281654 | Polyphenols |
|  | Illicium verum | salicylic acid | 338 | Polyphenols |
|  |  | shikimic acid | 8742 | Polyphenols |
|  | Origanum vulgare | 4- terpineol | 11230 | Terpenoids |
|  |  | Borneol | 64685 | Terpenoids |
|  |  | GAMMA-TERPINENE | 7461 | Terpenoids |
|  |  | Geraniol | 637566 | Terpenoids |
|  |  | germacrene d | 5317570 | Terpenoids |
|  |  | LINALYL ACETATE | 8294 | Terpenoids |
|  |  | THUJENE | 520384 | Terpenoids |
|  | Piper longum | Laurotetanine | 267400 | Alkaloid |
|  |  | Piperlonguminine | 5320621 | Alkaloid |
|  |  | Piplartine | 637858 | Alkaloid |
|  |  | sesamin | 72307 | phenol |
|  |  | Tetrahydropiperine | 581676 | Alkaloid |
|  | Trigonella foenum graceum | zingerone | 31211 | Polyphenols |
|  |  | Trigonelline | 5570 | Alkaloid |
|  |  | Trigoforin | 12267346 | Polyphenols |
|  |  | smilagenin | 91439 | Terpenoids |
|  |  | Scopoletin | 5280460 | Polyphenols |
|  |  | rhapontin | 637213 | Polyphenols |
|  |  | Medicarpin | 73067 | Polyphenols |
|  |  | Maackiain | 91510 | Polyphenols |
|  |  | Isovitexin | 162350 | Polyphenols |
|  |  | Hymecromone | 5280567 | Polyphenols |
|  |  | Gitogenin | 441887 | Terpenoids |
|  |  | gamma-Cadinene | 15094 | Terpenoids |
|  |  | DIOSGENIN | 99474 | Terpenoids |
|  |  | alpha-Muurolene | 12306047 | Terpenoids |
| Herbs | Acacia Catechu | AFZELECHIN | 42154 | Polyphenols |
|  |  | caffeic acid | 689043 | Polyphenols |
|  |  | Lupeol | 259846 | Terpenoids |
|  |  | Poriferasterol | 5281330 | phytosterol |
|  |  | protocatechuic acid | 72 | Polyphenols |
|  |  | quercitrin | 5280459 | Polyphenols |
|  |  | RUTIN | 5280805 | Polyphenols |
|  |  | TAXIFOLIN | 439533 | Polyphenols |
|  | Aconitum heterophyllum | Atisine | 441709 | Alkaloid |
|  |  | Heteratisine | 73527 | Alkaloid |
|  |  | Napelline | 441749 | Alkaloid |
|  | Adhatoda Vasica | Astragalin | 5282102 | Polyphenols |
|  |  | Peganine | 72610 | Alkaloid |
|  |  | Vasicinol | 442934 | Alkaloid |
|  |  | VASICINONE | 442935 | Alkaloid |
|  | Artemisia absinthium | Artemisinin | 68827 | Terpenoids |
|  |  | beta-Thujone | 91456 | Terpenoids |
|  |  | Bornyl acetate | 6448 | Terpenoids |
|  |  | Chamazulene | 10719 | Terpenoids |
|  |  | Guaiazulene | 3515 | Terpenoids |
|  |  | Myrcene | 31253 | Terpenoids |
|  | Clerodendron serratum | phytol | 5280435 | Terpenoids |
|  |  | Scutellarein | 5281697 | Polyphenols |
|  |  | squalene | 638072 | Terpenoids |
|  | Emblica officinalis | ellagic acid | 5281855 | Polyphenols |
|  | Glycyrrhiza glabra | 4'-O-Methylglabridin | 5319664 | Polyphenols |
|  |  | formononetin | 5280378 | Polyphenols |
|  |  | Glabridin | 124052 | Polyphenols |
|  |  | Hemileiocarpin | 70995758 | Polyphenols |
|  |  | Hispaglabridin B | 15228661 | Polyphenols |
|  |  | Licochalcone C | 9840805 | Polyphenols |
|  | Ocimum basilicum | Aromadendrene | 91354 | Terpenoids |
|  |  | Camphor | 2537 | Terpenoids |
|  |  | Eucalyptol | 2758 | Terpenoids |
|  |  | gamma-Muurolene | 6432308 | Terpenoids |
|  |  | tau-Cadinol | 160799 | Terpenoids |
|  | Ocimum sanctum | Carnosic acid | 65126 | Terpenoids |
|  |  | Myrtenal | 61130 | Terpenoids |
|  |  | rosmarinic acid | 5281792 | Polyphenols |
|  | Salvadora persica | D-Limonene | 440917 | Terpenoids |
|  |  | Pericalline | 6436240 | Alkaloid |
|  |  | Salvadoricine | 594163 | Alkaloid |
|  | salvia officinalis | Ferruginol | 442027 | Polyphenols |
|  |  | gamma-Elemene | 6432312 | Terpenoids |
|  |  | Genkwanin | 5281617 | Polyphenols |
|  |  | Hispidulin | 5281628 | Polyphenols |
|  |  | Sabinene | 18818 | Terpenoids |
|  |  | alpha-Cadinene | 12306048 | Terpenoids |
|  |  | alpha-Thujone | 261491 | Terpenoids |
|  |  | beta-Ocimene | 18756 | Terpenoids |
|  |  | Carnosol | 442009 | Terpenoids |
|  |  | Cirsimaritinv | 188323 | Polyphenols |
|  | Tinospora cardifolia | Columbin | 442015 | Terpenoids |
|  |  | Cordioside | 101915817 | Terpenoids |
|  |  | Ecdysterone | 5459840 | Terpenoids |
|  |  | Jatrorrhizine | 72323 | Alkaloid |
|  |  | magnoflorine | 73337 | Alkaloid |
|  |  | Palmatine | 19009 | Alkaloid |
|  |  | Tembetarine | 167718 | Alkaloid |
|  |  | tetrahydropalmatine | 5417 | Alkaloid |
|  | Zinziber officinale | 10-Gingerdione | 5317591 | Polyphenols |
|  |  | Ar-Curcumene | 3083834 | Terpenoids |
|  |  | beta-Bisabolene | 10104370 | Terpenoids |
|  |  | beta-Sesquiphellandrene | 12315492 | Terpenoids |
|  |  | Gingerenone A | 5281775 | Polyphenols |
|  |  | Paradol | 94378 | Polyphenols |
|  |  | Shogaol | 5281794 | Polyphenols |
|  |  | Tetrahydrocurcumin | 124072 | Polyphenols |
|  |  | (-)-Zingiberene | 521253 | Terpenoids |
| Others | Azadhirachta indica | gedunin | 12004512 | Terpenoids |
|  |  | Mahmoodin | 126566 | Terpenoids |
|  |  | Margolonone | 189726 | Terpenoids |
|  |  | Nimbidin | 108058 | Terpenoids |
|  |  | Nimbolide | 12313376 | Terpenoids |
|  | Camellia sinensis | Gallocatechin | 65084 | Polyphenols |
|  |  | Galloylquinic acid | 129650210 | Polyphenols |
|  |  | Theaflavine | 135403798 | Polyphenols |
|  |  | Catechin gallate | 6419835 | Polyphenols |
|  | ceratonia siliqua | Isolariciresinol | 160521 | Polyphenols |
|  |  | Methyl gallate | 7428 | Polyphenols |
|  |  | Pinoresinol | 73399 | Polyphenols |
|  | Zizipus spinachristi | Catechol | 289 | Polyphenols |
|  |  | Syringic acid | 10742 | Polyphenols |

**Table S3.** List of virtual screened phytochemical compounds against pantothenate synthetase protein of *Mycobacterium tuberculosis*.

| **S. no.** | **Ligand** | **Binding Affinity** |
| --- | --- | --- |
| 1 | 5280805 | -11 |
| 2 | 135403798 | -10.6 |
| 3 | 72307 | -10.5 |
| 4 | 107876 | -10.4 |
| 5 | 6419835 | -10.3 |
| 6 | 91439 | -10.2 |
| 7 | 441887 | -10.2 |
| 8 | 99474 | -10.2 |
| 9 | 5319664 | -10.2 |
| 10 | 124024 | -10.2 |
| 11 | 65576 | -10.1 |
| 12 | 73170 | -10 |
| 13 | 124052 | -9.9 |
| 14 | 15228661 | -9.9 |
| 15 | 441709 | -9.8 |
| 16 | 5154 | -9.7 |
| 17 | 53356215 | -9.7 |
| 18 | 5770 | -9.6 |
| 19 | 12004512 | -9.5 |
| 20 | 5281792 | -9.4 |
| 21 | 442015 | -9.4 |
| 22 | 12313376 | -9.4 |
| 23 | 73399 | -9.4 |
| 24 | 442021 | -9.4 |
| 25 | 441071 | -9.4 |
| 26 | 99615 | -9.4 |
| 27 | 5351516 | -9.4 |
| 28 | 64945 | -9.3 |
| 29 | 73527 | -9.2 |
| 30 | 101915817 | -9.2 |
| 31 | 159287 | -9.2 |
| 32 | 441774 | -9.2 |
| 33 | 5280343 | -9.2 |
| 34 | 10494 | -9.2 |
| 35 | 259846 | -9.1 |
| 36 | 439533 | -9.1 |
| 37 | 9840805 | -9.1 |
| 38 | 1794427 | -9.1 |
| 39 | 107971 | -9.1 |
| 40 | 441773 | -9.1 |
| 41 | 108058 | -9 |
| 42 | 72276 | -9 |
| 43 | 128861 | -9 |
| 44 | 107905 | -9 |
| 45 | 72277 | -9 |
| 46 | 5280445 | -9 |
| 47 | 64971 | -9 |
| 48 | 162350 | -8.9 |
| 49 | 5281855 | -8.9 |
| 50 | 65084 | -8.9 |
| 51 | 9064 | -8.9 |
| 52 | 5284649 | -8.9 |
| 53 | 5280863 | -8.9 |
| 54 | 5281654 | -8.8 |
| 55 | 442009 | -8.8 |
| 56 | 126566 | -8.8 |
| 57 | 101252065 | -8.7 |
| 58 | 101252066 | -8.7 |
| 59 | 637213 | -8.7 |
| 60 | 5280459 | -8.7 |
| 61 | 441749 | -8.7 |
| 62 | 5281697 | -8.7 |
| 63 | 5281617 | -8.7 |
| 64 | 5459840 | -8.7 |
| 65 | 65064 | -8.7 |
| 66 | 440735 | -8.7 |
| 67 | 5280961 | -8.7 |
| 68 | 53356107 | -8.7 |
| 69 | 5282102 | -8.6 |
| 70 | 70995758 | -8.6 |
| 71 | 5281628 | -8.6 |
| 72 | 6167 | -8.6 |
| 73 | 5280443 | -8.6 |
| 74 | 5471349 | -8.6 |
| 75 | 5281330 | -8.5 |
| 76 | 188323 | -8.5 |
| 77 | 189726 | -8.5 |
| 78 | 5280899 | -8.5 |
| 79 | 1550607 | -8.5 |
| 80 | 5281616 | -8.5 |
| 81 | 440832 | -8.5 |
| 82 | 5280442 | -8.4 |
| 83 | 267400 | -8.4 |
| 84 | 442154 | -8.4 |
| 85 | 73337 | -8.4 |
| 86 | 2353 | -8.4 |
| 87 | 72281 | -8.4 |
| 88 | 5280520 | -8.4 |
| 89 | 932 | -8.4 |
| 90 | 5320621 | -8.3 |
| 91 | 91510 | -8.3 |
| 92 | 5280378 | -8.3 |
| 93 | 167718 | -8.3 |
| 94 | 15376 | -8.3 |
| 95 | 5281243 | -8.3 |
| 96 | 5281607 | -8.3 |
| 97 | 53356108 | -8.3 |
| 98 | 68827 | -8.2 |
| 99 | 129650210 | -8.2 |
| 100 | 6419725 | -8.2 |
| 101 | 5280489 | -8.2 |
| 102 | 5281235 | -8.2 |
| 103 | 5281252 | -8.2 |
| 104 | 5280373 | -8.2 |
| 105 | 5317750 | -8.2 |
| 106 | 6436240 | -8.1 |
| 107 | 160521 | -8.1 |
| 108 | 446220 | -8.1 |
| 109 | 10219 | -8.1 |
| 110 | 101046549 | -8.1 |
| 111 | 5281708 | -8.1 |
| 112 | 637858 | -8 |
| 113 | 581676 | -8 |
| 114 | 65126 | -8 |
| 115 | 5281775 | -8 |
| 116 | 124072 | -8 |
| 117 | 638024 | -8 |
| 118 | 441074 | -8 |
| 119 | 3034034 | -8 |
| 120 | 54678486 | -8 |
| 121 | 72323 | -7.9 |
| 122 | 5288826 | -7.9 |
| 123 | 73067 | -7.8 |
| 124 | 442027 | -7.8 |
| 125 | 101744 | -7.7 |
| 126 | 5324625 | -7.7 |
| 127 | 445154 | -7.7 |
| 128 | 442934 | -7.6 |
| 129 | 19009 | -7.6 |
| 130 | 5417 | -7.6 |
| 131 | 90454 | -7.6 |
| 132 | 821448 | -7.6 |
| 133 | 442935 | -7.5 |
| 134 | 5317591 | -7.4 |
| 135 | 157301 | -7.4 |
| 136 | 5280460 | -7.3 |
| 137 | 10682896 | -7.3 |
| 138 | 5281794 | -7.2 |
| 139 | 12267346 | -7.1 |
| 140 | 5317570 | -7 |
| 141 | 5280567 | -7 |
| 142 | 2355 | -6.9 |
| 143 | 72610 | -6.9 |
| 144 | 94378 | -6.9 |
| 145 | 1742210 | -6.8 |
| 146 | 5281515 | -6.8 |
| 147 | 689043 | -6.8 |
| 148 | 6432308 | -6.8 |
| 149 | 3515 | -6.7 |
| 150 | 5281520 | -6.6 |
| 151 | 15094 | -6.6 |
| 152 | 12306047 | -6.6 |
| 153 | 91354 | -6.6 |
| 154 | 594163 | -6.6 |
| 155 | 445858 | -6.6 |
| 156 | 92231 | -6.5 |
| 157 | 9855795 | -6.5 |
| 158 | 19725 | -6.5 |
| 159 | 31211 | -6.5 |
| 160 | 72 | -6.5 |
| 161 | 638072 | -6.5 |
| 162 | 12315492 | -6.5 |
| 163 | 10719 | -6.4 |
| 164 | 160799 | -6.4 |
| 165 | 442793 | -6.4 |
| 166 | 637542 | -6.4 |
| 167 | 637566 | -6.3 |
| 168 | 12306048 | -6.3 |
| 169 | 18756 | -6.3 |
| 170 | 7428 | -6.2 |
| 171 | 8468 | -6.1 |
| 172 | 91456 | -6.1 |
| 173 | 6432312 | -6.1 |
| 174 | 10104370 | -6.1 |
| 175 | 10742 | -6.1 |
| 176 | 2519 | -6.1 |
| 177 | 370 | -6.1 |
| 178 | 1491 | -6.1 |
| 179 | 10364 | -6.1 |
| 180 | 261491 | -6 |
| 181 | 10442282 | -6 |
| 182 | 8742 | -5.9 |
| 183 | 6448 | -5.9 |
| 184 | 326 | -5.8 |
| 185 | 10281 | -5.8 |
| 186 | 338 | -5.8 |
| 187 | 5280435 | -5.8 |
| 188 | 61130 | -5.8 |
| 189 | 521253 | -5.8 |
| 190 | 9294 | -5.8 |
| 191 | 89594 | -5.8 |
| 192 | 3314 | -5.8 |
| 193 | 445070 | -5.8 |
| 194 | 6989 | -5.8 |
| 195 | 86608 | -5.7 |
| 196 | 11230 | -5.7 |
| 197 | 5570 | -5.7 |
| 198 | 440917 | -5.7 |
| 199 | 1183 | -5.7 |
| 200 | 7463 | -5.6 |
| 201 | 3083834 | -5.6 |
| 202 | 22311 | -5.6 |
| 203 | 637511 | -5.6 |
| 204 | 7127 | -5.5 |
| 205 | 11142 | -5.5 |
| 206 | 14896 | -5.5 |
| 207 | 7460 | -5.5 |
| 208 | 7461 | -5.5 |
| 209 | 8294 | -5.5 |
| 210 | 14525 | -5.4 |
| 211 | 16741 | -5.4 |
| 212 | 2346 | -5.4 |
| 213 | 8815 | -5.3 |
| 214 | 289 | -5.3 |
| 215 | 2537 | -5.2 |
| 216 | 2758 | -5.2 |
| 217 | 18818 | -5.2 |
| 218 | 6616 | -5.1 |
| 219 | 6549 | -5.1 |
| 220 | 64685 | -5.1 |
| 221 | 7673 | -5.1 |
| 222 | 121922 | -5 |
| 223 | 31253 | -5 |
| 224 | 7674 | -4.9 |
| 225 | 6654 | -4.9 |
| 226 | 520384 | -4.9 |
| 227 | 9985 | -4.7 |
| 228 | 5386591 | -4.7 |
| 229 | 206035 | -4.7 |
| 230 | 5350 | -4.2 |
| 231 | 65036 | -4.1 |
| 232 | 9794659 | -4.1 |
| 233 | 16315 | -3.9 |
| 234 | 206037 | -3.9 |
| 235 | 78160 | -3.8 |
| 236 | 16590 | -3.7 |
| 237 | 11617 | -3.7 |
| 238 | 5971 | -3.7 |
| 239 | 6324 | -1.8 |

**Table S4.** List of top ten virtually screened phytochemical compounds against pantothenate synthetase protein of *Mycobacterium tuberculosis* with their redocking result.

| **Compounds Pubchem ID** | **Phytochemical name** | **VS Result** | **Redocking Result** |
| --- | --- | --- | --- |
| 5280805 | **Rutin** | -11 | -11 |
| 135403798 | Theaflavine | -10.6 | -10.1 |
| 72307 | **Sesamin** | -10.5 | -10.5 |
| 107876 | Proanthocyanidins | -10.4 | -10 |
| 6419835 | **Catechin gallate** | -10.3 | -10.3 |
| 91439 | smilagenin | -10.2 | -10.2 |
| 441887 | Gitogenin | -10.2 | -10.2 |
| 99474 | DIOSGENIN | -10.2 | -10.2 |
| 5319664 | 4'-O-Methylglabridin | -10.2 | -10.2 |
| 124024 | 1,2,6-tri-O-galloyl-beta-d-glucopyranose | -10.2 | -10.2 |

**Table S5**: ADME analysis for the top three selected natural compounds as inhibitor against pantothenate synthetase protein.

| **Properties** | **Rutin** | **Sesamin** | **Catechin gallate** |
| --- | --- | --- | --- |
| iLOGP | 1.58 | 3.46 | 1.44 |
| XLOGP3 | -0.33 | 2.68 | 1.53 |
| WLOGP | -1.69 | 2.57 | 2.2 |
| MLOGP | -3.89 | 1.98 | 0.05 |
| Silicos-IT Log P | -2.11 | 3.25 | 1.04 |
| Consensus Log P | -1.29 | 2.79 | 1.25 |
| ESOL Log S | -3.3 | -3.93 | -3.7 |
| ESOL Solubility (mg/ml) | 3.08E-01 | 4.12E-02 | 8.85E-02 |
| ESOL Solubility (mol/l) | 5.05E-04 | 1.16E-04 | 2.00E-04 |
| ESOL Class | Soluble | Soluble | Soluble |
| Ali Log S | -4.87 | -3.5 | -4.86 |
| Ali Solubility (mg/ml) | 8.30E-03 | 1.13E-01 | 6.12E-03 |
| Ali Solubility (mol/l) | 1.36E-05 | 3.20E-04 | 1.38E-05 |
| Ali Class | Moderately soluble | Soluble | Moderately soluble |
| Silicos-IT LogSw | -0.29 | -4.6 | -3.09 |
| Silicos-IT Solubility (mg/ml) | 3.15E+02 | 8.98E-03 | 3.60E-01 |
| Silicos-IT Solubility (mol/l) | 5.15E-01 | 2.54E-05 | 8.15E-04 |
| Silicos-IT class | Soluble | Moderately soluble | Soluble |
| GI absorption | Low | High | Low |
| BBB permeant | No | Yes | No |
| Pgp substrate | Yes | No | No |
| CYP1A2 inhibitor | No | No | No |
| CYP2C19 inhibitor | No | Yes | No |
| CYP2C9 inhibitor | No | No | No |
| CYP2D6 inhibitor | No | Yes | No |
| CYP3A4 inhibitor | No | Yes | No |
| log Kp (cm/s) | -10.26 | -6.56 | -7.91 |
| Lipinski #violations | 3 | 0 | 1 |
| Ghose #violations | 4 | 0 | 0 |
| Veber #violations | 1 | 0 | 1 |
| Egan #violations | 1 | 0 | 1 |
| Muegge #violations | 4 | 0 | 2 |
| Bioavailability Score | 0.17 | 0.55 | 0.55 |
| PAINS #alerts | 1 | 0 | 1 |
| Brenk #alerts | 1 | 0 | 1 |
| Leadlikeness #violations | 1 | 1 | 1 |
| Synthetic Accessibility | 6.52 | 4.12 | 4.16 |

**Table S6**. Calculated net Binding free energy and energy components values for pantothenate synthetase protein complexes with phytochemical compounds snapshots collected from last 10 ns MD simulation trajectories.

| **Energy**  **components** | **Rutin** | **Sesamin** | **Catechin gallate** | **Diphosphomethylphosphonic acid adenosylester** |
| --- | --- | --- | --- | --- |
| **∆G Bind** | -82.24±9.35 | -69.78±3.88 | -66.83±4.5 | -96.44±12.82 |
| **∆G Bind Coulomb** | -55.41±7.96 | -15.63±2.37 | -127.95±13.58 | -303.34±101.39 |
| **∆G Bind Covalent** | 6.42±1.57 | 1.78±0.3 | 6.71±1.76 | 3.267±1.54 |
| **∆G Bind Hbond** | -6.21±0.65 | -1.13±0.29 | -4.96±0.42 | -9.69±0.71 |
| **∆G Bind Lipo** | -11.54±2.49 | -20.87±0.75 | -13.49±1.05 | -5.45±0.59 |
| **∆G Bind Packing** | -2.42±0.56 | -1.06±0.39 | -2.47±0.36 | -0.29±0.17 |
| **∆G Bind Solv GB** | 56.13±3.37 | 21.60±1.92 | 133.53±12.58 | 257.23±86.37 |
| **∆G Bind vdW** | -69.20±3.74 | -54.47±1.8 | -58.19±1.77 | -38.14±5.15 |
| **Lig Strain Energy** | 10.39±2.33 | 3.20±0.27 | 6.91±2.16 | 5.92±2.79 |

**Additional Data**

Drug-likeness evaluation of all 239 phytochemical compounds screened against pantothenate synthetase protein.

**Figure S1.**


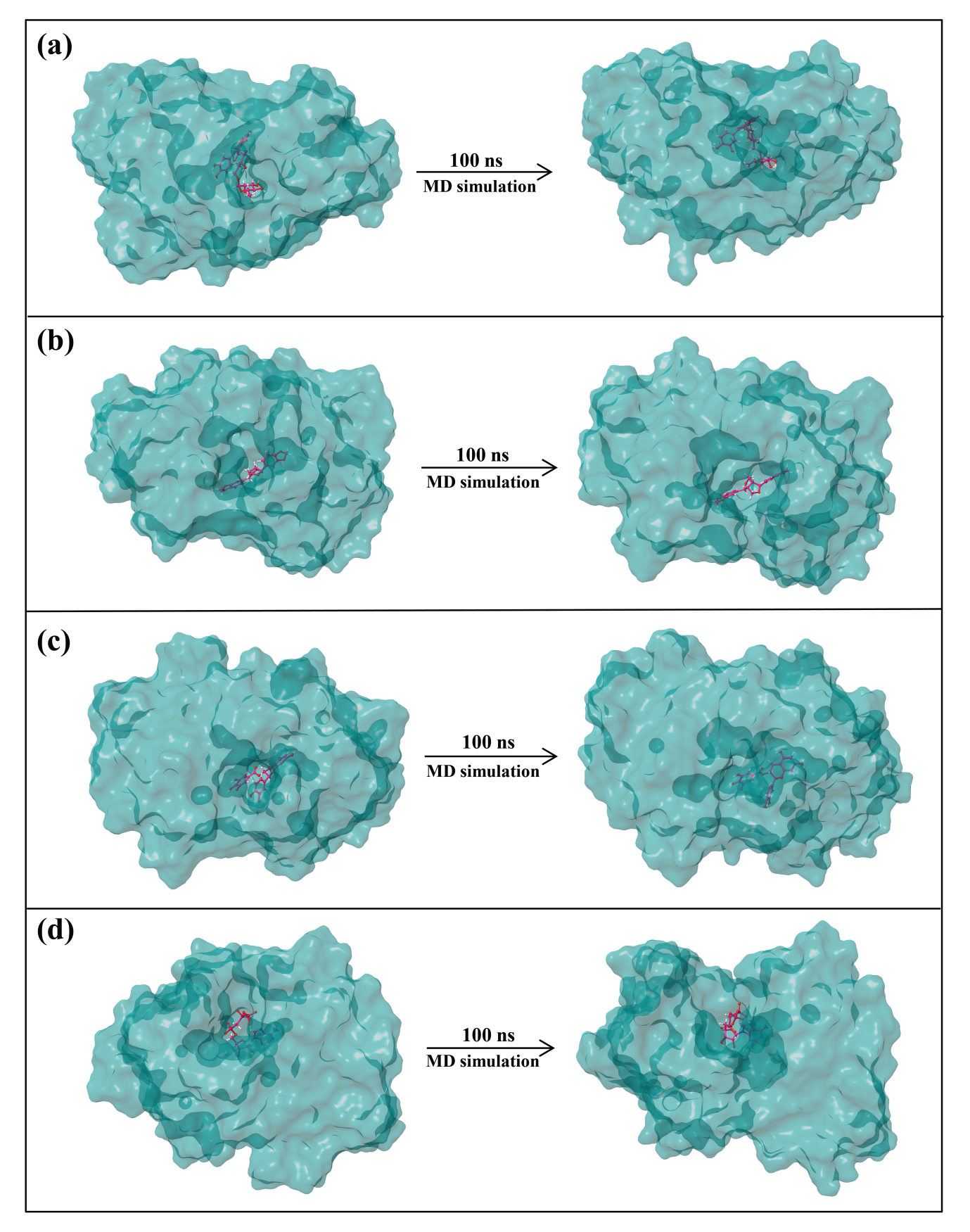


**Figure S1.** 3D surface conformational changes between the first pose and last pose, i.e. (a) Rutin, (b) Sesamin, (c) Catechin gallate and (d) Diphosphomethylphosphonic acid adenosylester reference molecule, extracted from the 100 ns MD simulation trajectories.

**Figure S2.**


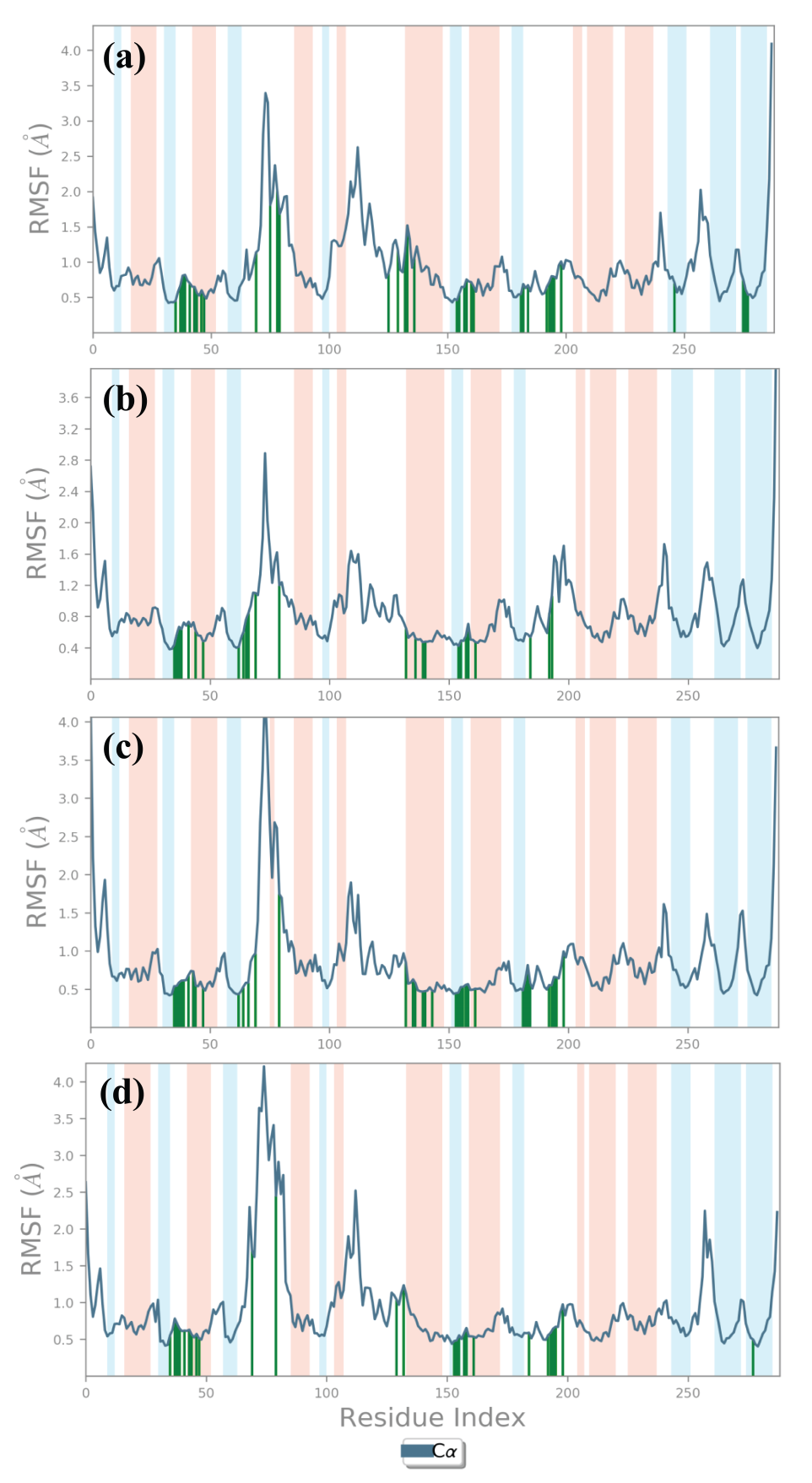


**Figure S2.** RMSF plot generated for the pantothenate synthetase protein docked with selected phytochemical compounds and reference molecule, i.e., (a) Rutin, (b) Sesamin, (c) Catechin gallate and (d) Diphosphomethylphosphonic acid adenosylester.

**Figure S3.**


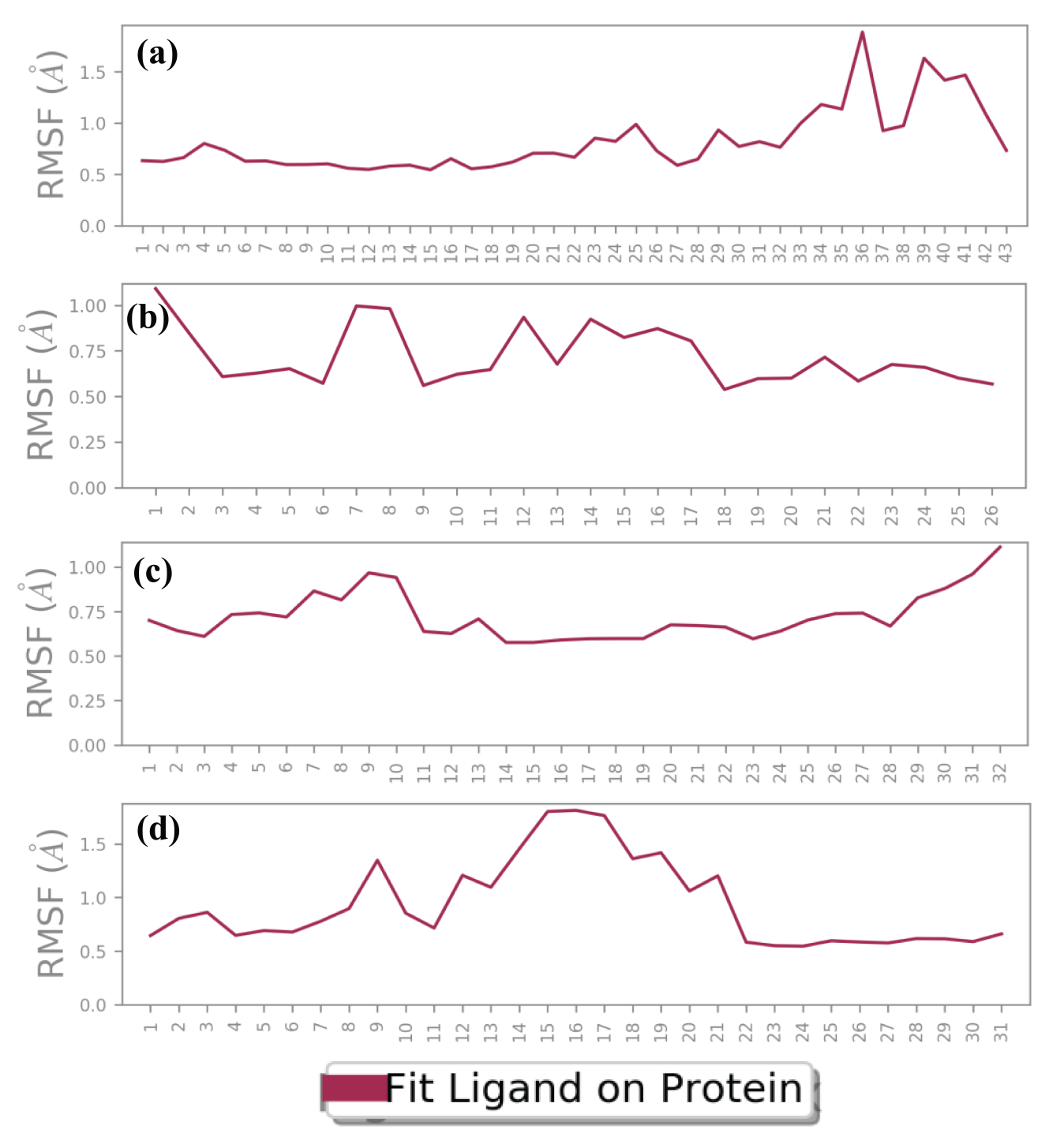


**Figure S3.** RMSF plot generated for the docked phytochemical compounds and reference molecule, i.e., (a) Rutin, (b) Sesamin, (c) Catechin gallate and (d) Diphosphomethylphosphonic acid adenosylester, fit in the pantothenate synthetase protein during 100 ns molecular dynamics simulation interval.

**Figure S4.**

**
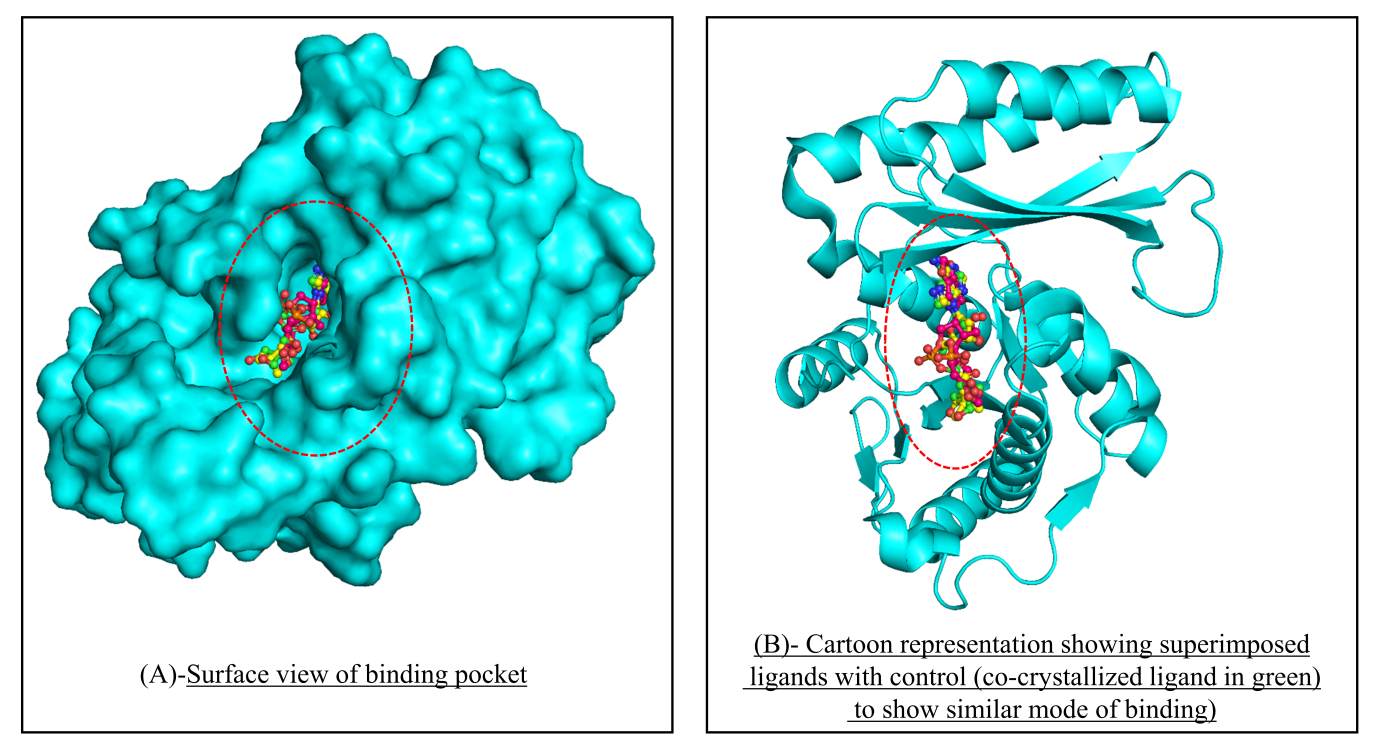
**

**Figure S4. (**A) Surface view of binding pocket, (B) Cartoon representation showing superimposed ligands with control (co-crystallized ligand in green) to show similar mode of binding).

**Figure S5**

**
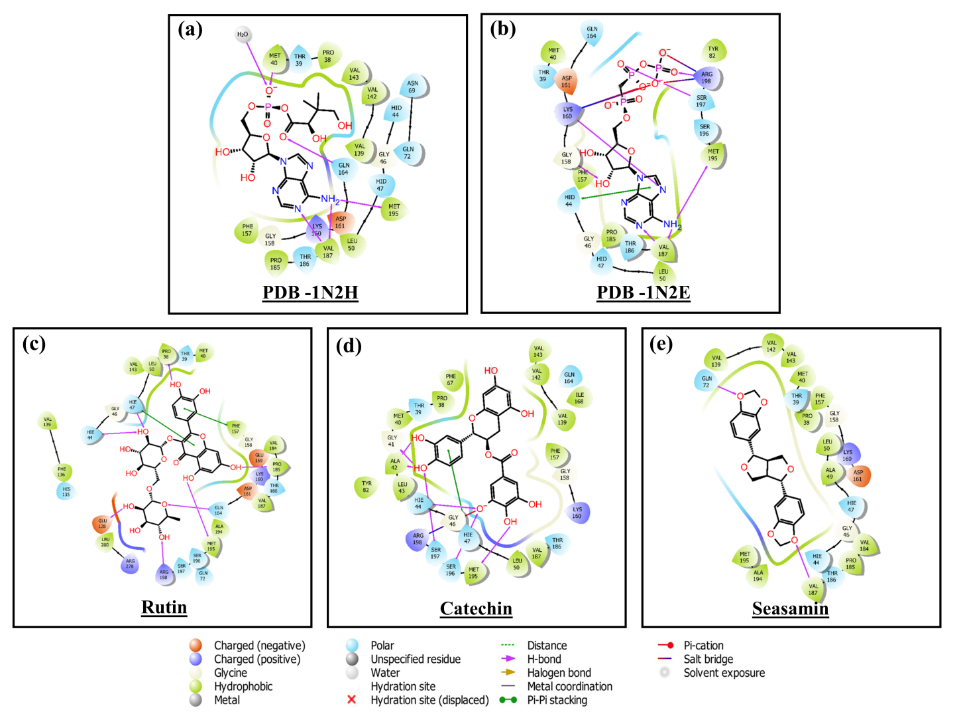
**

**Figure S5.** The 2D interaction diagram showing the control inhibitor along with three lead molecules. The diagram illustrates the molecular interactions and contacts between each molecule and its target.

**Figure S6.**


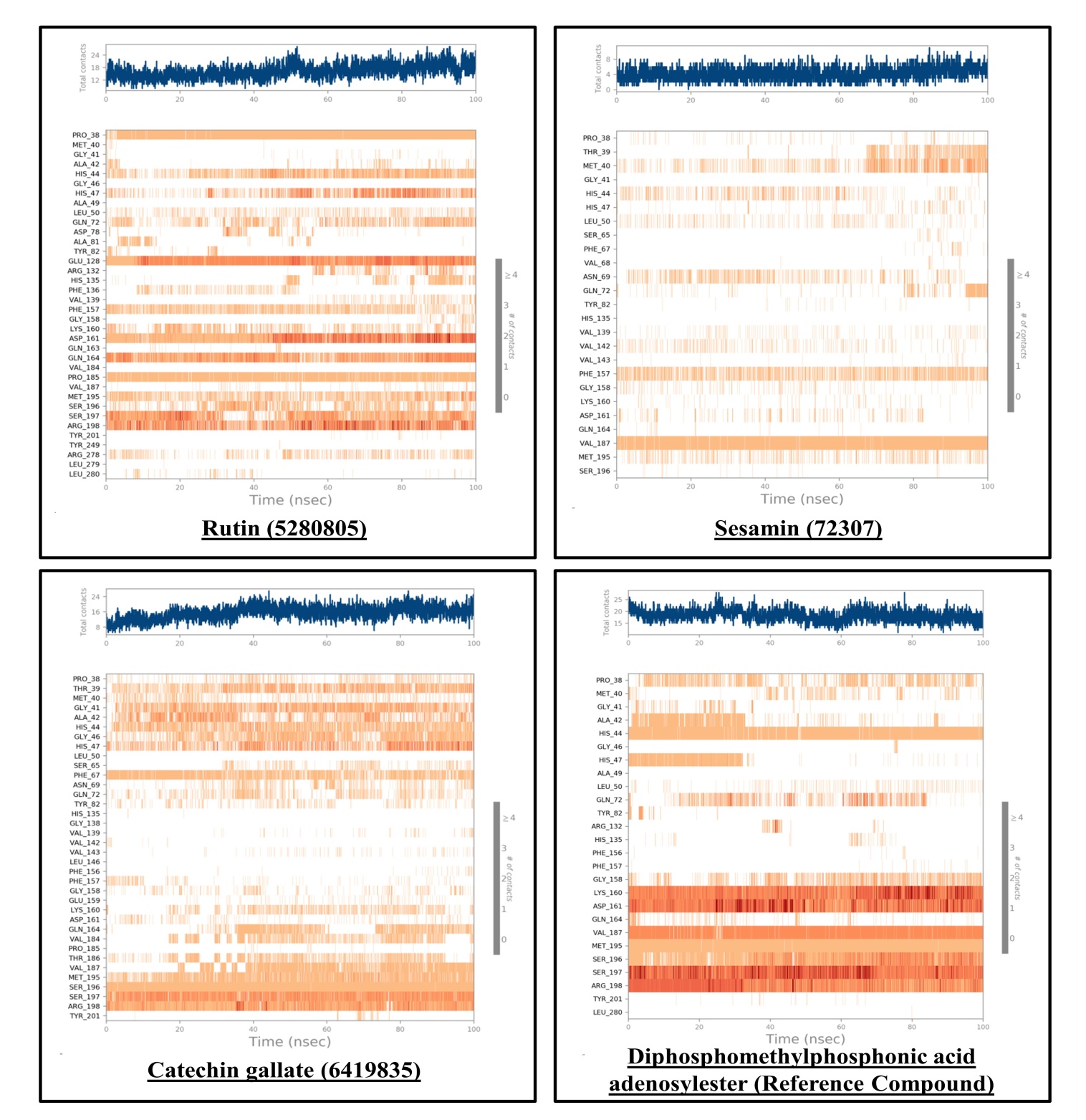


**Figure S6.** A timeline representation of interactions and contacts. The top panel displays total specific contacts between protein and ligand over the whole MD simulation trajectory. The bottom panel indicates residues interacting with the ligand in each frame, with darker shades representing multiple contacts.
